# Engineering single-molecule fluorescence with asymmetric nano-antennas

**DOI:** 10.1038/s41377-021-00522-9

**Published:** 2021-04-14

**Authors:** Wenqi Zhao, Xiaochaoran Tian, Zhening Fang, Shiyi Xiao, Meng Qiu, Qiong He, Wei Feng, Fuyou Li, Yuanbo Zhang, Lei Zhou, Yan-Wen Tan

**Affiliations:** 1 State Key Laboratory of Surface Physics and Department of Physics, Fudan University, Shanghai, 200433 China; 2 Shanghai Institute for Advanced Communication and Data Science, Shanghai University, Shanghai, 200444 China; 3 Key Laboratory of Specialty Fiber Optics and Optical Access Networks, Joint International Research Laboratory of Specialty Fiber Optics and Advanced Communication, Shanghai University, Shanghai, 200444 China; 4 Department of Chemistry and State Key Laboratory of Molecular Engineering of Polymers, Fudan University, Shanghai, 200433 China; 5 Institute for Nanoelectronic Devices and Quantum Computing, Fudan University, Shanghai, 200433 China; 6 Collaborative Innovation Center of Advanced Microstructures, Nanjing, 210093 China; 7 Multiscale Research Institute of Complex Systems, Fudan University, Shanghai, 200433 China

**Keywords:** Nanophotonics and plasmonics, Nanophotonics and plasmonics

## Abstract

As a powerful tool for studying molecular dynamics in bioscience, single-molecule fluorescence detection provides dynamical information buried in ensemble experiments. Fluorescence in the near-infrared (NIR) is particularly useful because it offers higher signal-to-noise ratio and increased penetration depth in tissue compared with visible fluorescence. The low quantum yield of most NIR fluorophores, however, makes the detection of single-molecule fluorescence difficult. Here, we use asymmetric plasmonic nano-antenna to enhance the fluorescence intensity of AIEE1000, a typical NIR dye, by a factor up to 405. The asymmetric nano-antenna achieve such an enhancement mainly by increasing the quantum yield (to ~80%) rather than the local field, which degrades the molecules’ photostability. Our coupled-mode-theory analysis reveals that the enhancements stem from resonance-matching between antenna and molecule and, more importantly, from optimizing the coupling between the near- and far-field modes with designer asymmetric structures. Our work provides a universal scheme for engineering single-molecule fluorescence in the near-infrared regime.

## Abstract (web summary)

This work achieves drastic fluorescence enhancement of NIR single-molecule based on asymmetric plasmonic nano-antenna, by fine-tuning the near- and far- field properties of plasmonic mode.

## Graphical abstract

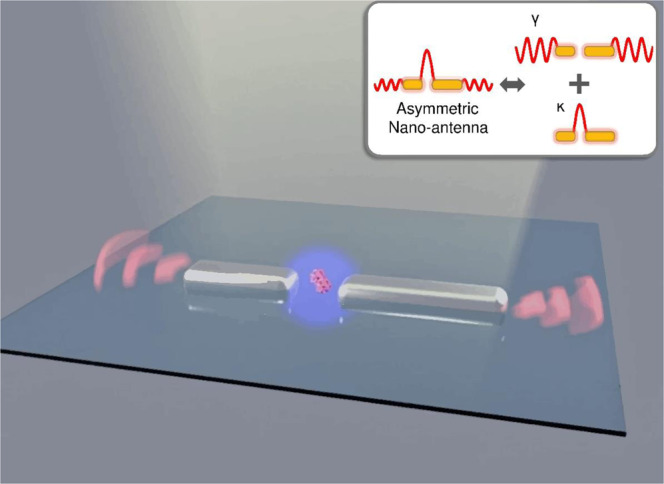

## Introduction

Single-molecule fluorescence detection (SMFD) is able to probe, one molecule at a time, dynamical processes that are crucial for understanding functional mechanisms in biosystems^1–3^. Signal to noise ratio (SNR) is a critical factor determining the temporal and spatial resolutions in SMFD. Fluorescence in the Near-infrared (NIR) offers improved SNR by reducing the scattering, absorption, and autofluorescence from biological cellular or tissue samples. NIR fluorescence, therefore, provides high imaging resolution with increased tissue penetration depth that are important for biomedical applications^4^. However, most NIR-emitters suffer from low quantum yield (denoted by Φ)^4,5^; the weak NIR fluorescence signal makes the detection extremely difficult.

Plasmonic nanostructures are capable of converting localized electromagnetic energy into free radiation and vice versa^6^. This capability makes them efficient nano-antennas for modulating molecular fluorescence^7–12^. The plasmonic nano-antenna generally enhances the fluorescence of a nearby molecule by enhancing the excitation rate and the quantum yield of the molecule. The total fluorescence enhancement *F*_*E*_ is the product of the two enhancement factors, *F*_*E*_ = *F*_*exc*_ · *F*_*em*_, where *F*_*exc*_ denotes the enhancement on excitation rate while *F*_*em*_ (defined as: $F_{em} = \Phi /\Phi _0$ where Φ_0_ is the quantum yield of the isolated molecule) denotes the enhancement on quantum yield of the molecule. A common practice trying to increase *F*_*E*_ has been tuning the resonances of the antenna to meet one (or both) of the excitation/emission frequencies of the molecules^7–12^. However, the practice does not usually guarantee an optimized *F*_*E*_; to achieve large *F*_*E*_, it is imperative that both *F*_*exc*_ and *F*_*em*_ are optimized for a particular antenna design. Such optimization processes for *F*_*exc*_ and *F*_*em*_ require different (sometimes conflicting) properties of the plasmonic modes in the antenna. Carefully balancing the conflicting requirements to achieve optimal *F*_*E*_ is a central issue in designing plasmonic nano-antenna. Specifically, in order to optimally enhance the fluorescence, the plasmonic mode of the nano-antenna has to (1) couple strongly to the molecule and (2) radiate strongly to free space. Simultaneously satisfying the two requirements poses a challenge that is impossible to overcome in conventional, symmetric plasmonic nanostructures.

Armed with this insight, here we solve the challenge by introducing additional tuning parameters—asymmetry of the nano-antenna in particular—to enhance NIR single-molecule dye’s fluorescence intensity. Specifically, we construct asymmetric nano-antennas consisting of two bars with unequal lengths that provide multiple plasmonic modes with tunable resonance frequencies matching both excitation and emission frequencies of the fluorophore. The added tuning parameter, i.e., the ratio of the bar lengths, in such asymmetric structures offers new possibilities to modulate the near-field and far-field properties of the plasmonic modes, thereby further improving both *F*_*exc*_ and *F*_*em*_. As a result, we acquire a single-molecule fluorescence enhancement factor up to 405, and the quantum yield extracted from our simulations reaches ~80%. Because the quantum yield plays a major role in this setup, we achieve this enhancement without sacrificing the molecules’ survival time under laser irradiation. Our work, therefore, establishes a novel, universal approach to enhance single-molecule fluorescence in the NIR regime without compromising the molecule’s photostability.

## Results

**Fig. 1:**
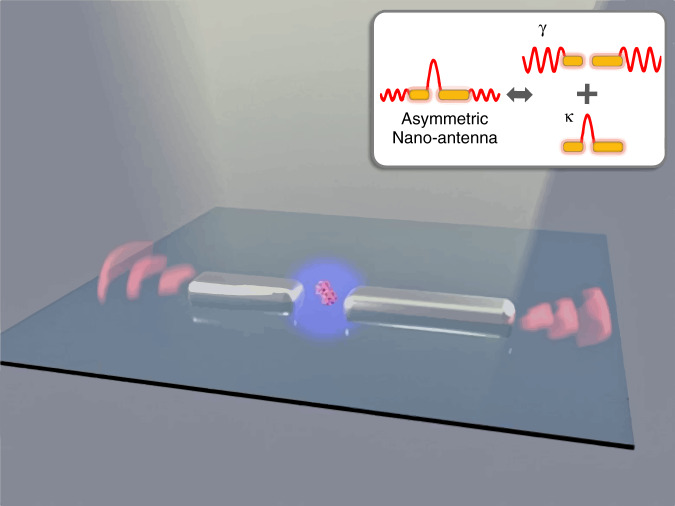
Scheme of the florescence enhancement enabled by nano-antenna system. Florescence of molecule-antenna system is determined by both near-field coupling efficiency from molecule to antenna (purple sphere indicates near-field distribution) and radiation capability from antenna to free space (red waves indicates far-field distribution). Red-helix is molecule placed at the gap of antenna, yellow light beam represents the excitation illumination. Inset schematically depicts a typical plasmonic mode supported by our asymmetric nano-antenna, with far-field part determining the radiation capability and near-field part determining the molecule-antenna coupling

**Fig. 2:**
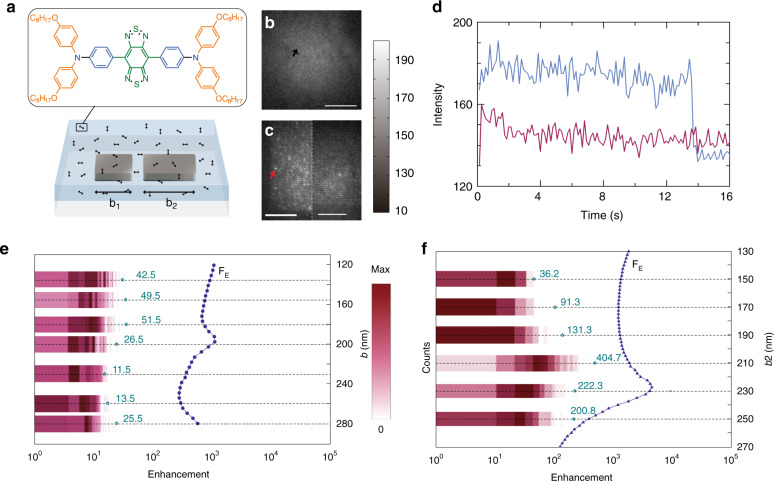
Experimental setup and measurement of F_E_ for single AIEE1000 molecule. **a** Schematic of double-bar nano-antenna (with bar length *b*_1_ and *b*_2_) coated with AIEE1000 molecules (black double ended arrows) in PMMA (light blue) on glass substrate (light gray). Inset shows the chemical structure of AIEE1000. **b** Fluorescence image of AIEE1000 in PMMA without antennas. Scale bar, 10 µm. Data are collected when excitation power density is 32 kW/cm^2^, and are rescaled for direct comparison with image in Fig. 2c. **c** Fluorescence image of asymmetric antenna array (left) and symmetric antenna array (right) coated with AIEE1000 in PMMA. Data are collected when excitation power density is 32 kW/cm^2^. Scale bar, 10 µm. **d** Fluorescence time traces of a single pixel as arrows indicated in **b** (red curve) and **c** (blue curve). **e** Histograms of F_E_ with symmetric double-bar antennas. Each histogram shows the distribution of fluorescence enhancement from molecules near symmetric double-bar antennas with bar-length ranging from 135 nm to 280 nm. Maximums of simulated F_E_ are plotted with blue dash-dotted lines. **f** Histograms of F_E_ with asymmetric double-bar antennas. Each histogram shows the distribution of fluorescence enhancement coming from molecules near asymmetric double-bar antenna with *b*_1_ fixed at 150 nm and *b*_2_ varying from 150 nm to 250 nm. Maximums of simulated FE are plotted with blue dash-dotted lines, consisting with corresponding experimental results. The histograms in **e**, **f** are normalized with the maximum counts of enhancement for each experiment case, where the exact number of “max” are totally different for each case

We start with AIEE1000, a typical donor-acceptor-donor organic dye with central emission wavelength ~1050 nm^13^. The molecule is suitable for labeling, and is metabolizable in organism; these features make it a promising probe in biomedical imaging and detection^13^. AIEE1000, like most other NIR-II dyes, has a quantum yield of only 1.19% (in polymethyl methacrylate, PMMA; Supplementary Information, Section 1) (Fig. 1). Our demonstration of fluorescence enhancement in AIEE1000 is, in principle, applicable to a wide range of NIR dyes. We fabricate two silver bars with equal or unequal length on a glass substrate, and use them as a plasmonic nano-antenna for fluorescence enhancement in AIEE1000 (see Methods; we chose silver because it has a relatively small intrinsic loss^14–16^.) The double-bar antennas are arranged in an array with 700 nm spacing between neighboring antennas (Fig. 2a). We then disperse AIEE1000 molecules in a PMMA matrix, and spin-coat the PMMA on top of the antenna array. (PMMA thickness ≈ 60 nm, AIEE1000: 10 µmol/L, around 10 molecules per pixel-area) Finally, we observe the fluorescence of AIEE1000 using total-internal-reflective-fluorescence microscopy under 633 nm laser excitation (Fig. 2b, c; see Methods and Section 2 of Supplementary Information for details).

The double-bar nano-antenna significantly enhances the fluorescence of single molecules in its vicinity. The enhancement allows us to observe, for the first time, the photo-bleach of a single AIEE1000 fluorescence molecule. Photo-bleach refers to the irreversible process that organic fluorophores are excited to a dark state after a certain number of excitation-emission cycles; a sudden drop in fluorescence intensity is the signature of the photo-bleach of a single fluorophore^3,12^. Without nano-antenna, such photobleaching events from single AIEE1000 molecules are too weak to be detected because of the molecules’ low quantum yield (red curve in Fig. 2d). The nano-antenna, however, makes the photo-bleach clearly visible in single AIEE1000 molecules placed close to the antennas (Fig. 2d, blue curve). We estimate the fluorescence enhancement factor from the ratio of fluorescence intensity (normalized to laser excitation power) with and without nano-antenna:

1 $$F_E = \frac{{I_a \cdot P_g}}{{P_a \cdot I_g}}$$

where *I*_*a*(*g*)_ is the single-molecule fluorescence signal with (without) nano-antenna, and *P*_*a*_ and *P*_*g*_ are laser excitation power in both cases. Here *I*_*a*_ can be directly obtained from the intensity drop caused by the photobleach; meanwhile we estimate *I*_*g*_ by calculating the increase rate of fluorescence when changing AIEE1000 concentration (see Methods and Section 2 of Supplementary Information). (To eliminate errors, our analyses only include single molecules both colocalized with antennas and displaying bleaching-steps.) Because of the random location and orientation of the molecules with respect to the antennas, the measured *F*_*E*_ exhibits certain distributions (Fig. 2e); only the maxima represent molecules located at the optimized hot spots of the antennas. We find the maximum *F*_*E*_ enabled by symmetric nano-antennas reaches 50 at a bar length of 180 nm.

Making the double-bar antenna asymmetric further enhance the fluorescence of single-molecule AIEE1000. Figure 2f displays the fluorescence enhancement of asymmetric double-bar antennas with the short bar fixed at 150 nm while the long bar changes from 150 nm to 250 nm (the gap between two bars is kept at 40 nm, the same gap size used in symmetric configurations). In contrast to symmetric antennas, the asymmetric antennas offer much higher single-molecule enhancements. The maximum *F*_*E*_ exceeds 100 in most cases under study, and peaks at 405 for antennas with the long bar length of 210 nm (Fig. 2f). We note that quantitative discrepancies exist between predicted and measured peak positions (see Fig. 2e, f), which can be attributed to the uncertainties in sample geometry, as well as discrete bar length *b*_2_, in our experiment.

**Fig. 3:**
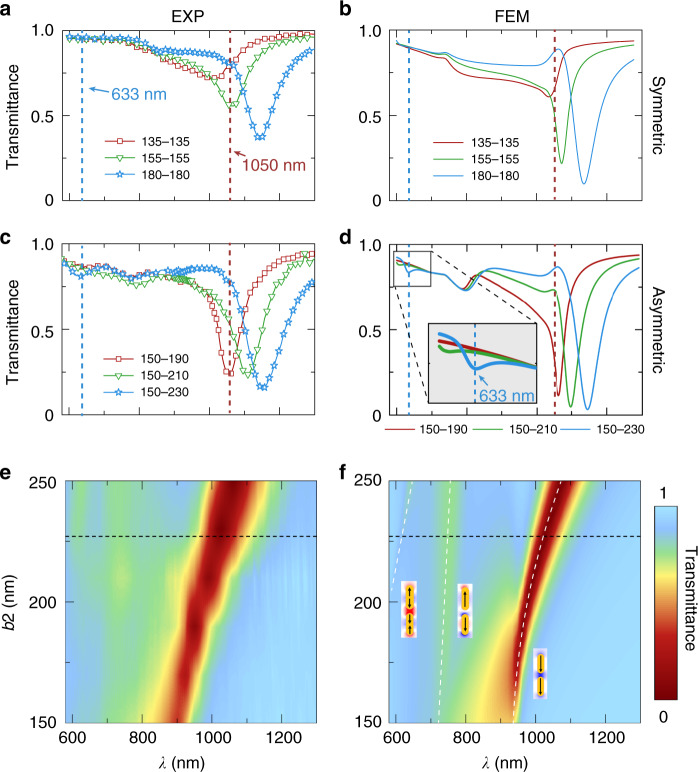
Measured and simulated transmission spectra of nano-antenna arrays. **a, b** Measured and simulated transmittance spectra of the symmetric samples. **c, d** Measured and simulated transmittance spectra of the asymmetric samples. **e, f** Measured and simulated transmittance versus *b*_2_ and wavelength for the asymmetric samples. From right to left, Fig. 3f insets shows the binding, anti-binding mode of dipolar resonance, and the binding mode of quadrupole mode. The anti-binding modes of quadrupole resonance are at wavelengths shorter than 600 nm (not shown). Black dash line denotes the position of *b*_2_ = 227 *nm*. White dashed lines denote the evolutions of resonant modes in such nano-antennas with insets schematically depicting the current distributions of the modes

How do we understand the large fluorescence enhancement induced by both symmetric and asymmetric double-bar nano-antennas? Important clues come from far-field characterization of the nano-antennas. Figure 3a, c display the transmittance spectra of three typical samples containing arrays of nano-antennas in symmetric and asymmetric configurations, respectively. Various plasmonic modes manifest as dips in the spectra. In particular, an evolving plasmonic mode near emission wavelength (1050 nm) signifies the existence of a plasmonic mode that is tuned by the bar length. In addition, the spectra of asymmetric antennas exhibit an additional dip in the vicinity of the excitation wavelength (633 nm), which is missing in the spectra of symmetric antennas (Fig. 3c).

Next, we employ finite-element-method (FEM) to decipher the role of various plasmonic modes in enhancing the fluorescence of single molecules. We first point out that the FEM simulation captures all salient features of the transmission spectra (Fig. 3b, d). In particular, the high-frequency mode observed in the vicinity of the excitation wavelength is clearly resolved in the simulation (Fig. 3d inset). More importantly, a detailed simulation immediately identifies the plasmonic modes responsible for the observed fluorescence enhancement. Figure 3f displays the evolution of the plasmonic modes as the length of the long bar is varied in the simulation (experimental data are shown in Fig. 3e). Because the nano-antenna at optimal enhancement supports just two plasmonic modes matching the emission and excitation wavelengths (Fig. 3e, f, black dashed line), the two modes are primarily responsible for the enhancement. Our FEM simulation shows that the mode pair in the emission regime are the binding and anti-binding modes of dipolar resonances of two bars, whereas those in the excitation regime are the binding and anti-binding modes of quadrupole resonances of two bars, respectively (Fig. 3f insets). We note that the anti-binding modes of quadrupole resonance are at wavelengths shorter than 600 nm, which are not shown in Fig. 3f. The simulation does not, however, capture all physics involved; for example, the mode-matching mechanism does not explain why a weak resonance at excitation wavelength enhances *F*_*E*_ in such a pronounced way (Fig. 2).

**Fig. 4:**
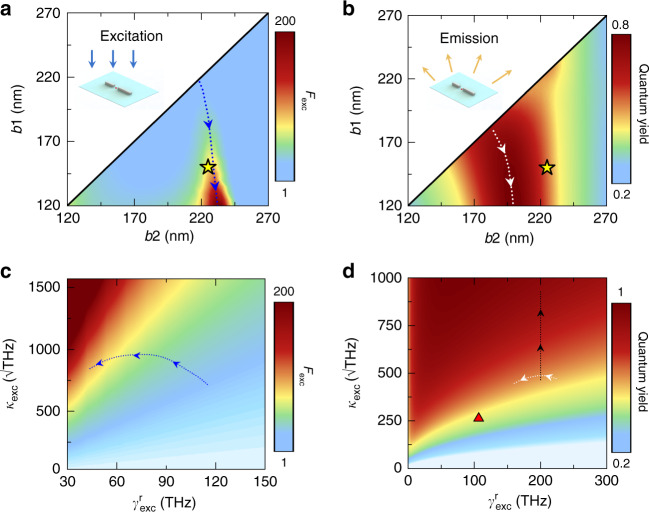
Phase diagrams and CMT analyses of the molecule-antenna system. Numerically computed with FEM simulations **a** excitation enhancement (F_exc_, calculated at 633 nm) and **b** quantum yield (calculated at 1050 nm) of the asymmetric antennas versus bar lengths *b*_1_ and *b*_2_. **c** Color map represents CMT-computed F_exc_ (at 633 nm) versus κ_exc_ and ${\upgamma}_{{\mathrm{exc}}}^{\mathrm{r}}$ with other CMT parameters fixed as: ${\mathrm{d}}_{{\mathrm{exc}}}^0 = 2.3\,\sqrt {{\mathrm{THz}}}$, ${\upgamma}_{{\mathrm{exc}}}^{\mathrm{i}} = 7.6\,{\mathrm{THz}}$, and the blue dashed line describes the evolution of those antennas located on the blue line in **a**. **d** Color map represents CMT-computed quantum yield (at 1050 nm) versus κ_exc_ and ${\upgamma}_{{\mathrm{exc}}}^{\mathrm{r}}$ with other CMT parameters fixed as ^∼^γ_nr_ = 272 THz, γ_em_^i^ = 1.3 THz, the white dashed line describes the evolution on those antennas located on the white dashed line in **b**, the black dashed line represents a series of antennas with gap distance decreasing from 80 nm to 10 nm and the red triangle represents an undesigned symmetric antenna (see Sec. 8 in SI for their geometric and CMT parameters)

To this end, we explore the underlying physics with FEM simulations on realistic molecule excitation/emission processes (see insets to Fig. 4a, b). Figure 4a, b depict how the antenna-enabled enhancements on excitation *F*_*exc*_ and quantum yield *Φ* vary against the bar length *b*_1_ and *b*_2_, computed by FEM simulations at 633 nm and 1050 nm, respectively (see simulation method in Sec. 5 of SI). In both figures, we find significant enhancement in regions centered at two dashed lines, representing the evolutions of nano-structures exhibiting plasmonic modes with resonance wavelengths hitting 633 nm (Fig. 4a) or 1050 nm (Fig. 4b), respectively. These results are consistent with the resonance-matching mechanism noted in previous studies^7–12^. However, within these frequency-matched enhancement bands, different antennas still behave distinctly, indicating that the properties of plasmonic modes play very important roles. Specifically, as the structural asymmetry of our nano-antenna increases (along the dashed lines following the arrows), both *F*_*exc*_ and *Φ* can be further enhanced. Enhancement on F_exc_ is more significant, simply because the antenna-enhanced *Φ* has already reached a value as high as ~0.8, leaving very little room for further enhancement on *F*_*exc*_. These two phase diagrams also explain why the experimentally identified optimized nano-antenna (with *b*_1_ = 150 nm, *b*_2_ = 210 nm) yield excellent enhancements, as its location (the yellow star) is very close to two enhancement regions in Fig. 4a, b. Finally, we perform full-wave simulations based on FEM to compute the fluorescence enhancements (see Sec. 5 in SI) with varying bar lengths in both symmetric and asymmetric configurations. Even though the simulated *F*_*E*_ is much higher than measured values, our simulated results (blue curves in Fig. 2e, f) have qualitatively reproduced salient features of experimental results. We attribute the discrepancy to the fact that it is difficult to precisely place our molecule at the bull’s eye of the “hot spot” as we did in our simulations.

To further understand why asymmetric nano-antennas perform better on enhancing *F*_*exc*_ and *ϕ* we use the coupled mode theory (CMT) to further analyze the excitation and emission processes^17^. Our CMT analyses (see Sec. 6 of SI for details) reveal that F_exc_ and *ϕ* are given by

2 $$F_{exc} = \frac{{\left| {d_{exc}^0} \right|^2}}{{\left| C \right|^2}}\frac{{\left| {\kappa _{exc}} \right|^2}}{{\left( {\gamma _{exc}^r + \gamma _{exc}^i} \right)^2}}$$

3 $$\phi = \left( {\frac{{\gamma _{em}^r}}{{\gamma _{em}^r + \gamma _{em}^i}}} \right)\left( {\frac{1}{{1 + \tilde \gamma _{nr}\frac{{\gamma _{em}^r + \gamma _{em}^i}}{{\kappa _{em}^2}}}}} \right)$$

under the excitation/emission resonance-matching condition. Here, *κ*_*exc*_(*κ*_*em*_), $\gamma _{exc}^r\left( {\gamma _{em}^r} \right)$, and $\gamma _{exc}^I(\gamma _{em}^i)$ represent, respectively, the molecule-antenna near-field coupling with the molecule and the radiation/absorption damping rates of the plasmonic mode responsible for excitation (emission) process, $d_{exc}^0$ denotes the coupling between incident wave and the excited mode, *C* is a dimensionless constant dictated by the substrate, and $\tilde \gamma _{nr}$ denotes the effective absorption damping of the molecule-antenna system. Equations (2–3) reveal that, apart from resonance-matching, fluorescence enhancement also sensitively depends on the properties of two plasmonic modes. In particular, the antenna-enhanced quantum yield contains two competing terms (see Eq. (3)). The first term describes the antenna’s efficiency (portion of radiation energy out of total energy absorbed by the antenna), and the second measures the molecule’s capability to transfer its energy to the plasmonic mode via near-field coupling. We find that all absorption-related parameters $\left( {{\mathrm{i}}.{\mathrm{e}}.,\gamma _{exc}^I,\gamma _{em}^I,\tilde \gamma _{nr}} \right)$are insensitive to structure changes, since they are mainly determined by the constitutional materials. Meanwhile, $d_{exc}^0$ is very small in all cases studied, quite insensitive to the structure changes (Supplementary Information Section 7). To capture the essential physics, we only consider how *F*_*exc*_ and *ϕ* depend on the near-field coupling (i.e., *κ*_*exc*_, *κ*_*em*_) and far-field radiation properties $\left( {{\mathrm{i}}.{\mathrm{e}}.,\,\gamma _{exc}^r,\gamma _{em}^r} \right)$ of the plasmonic modes responsible for the two processes. Color maps in Fig. 4c, d plot how *F*_*exc*_ and Φ vary against *κ* and *γ*^*r*^ of two “modes”, calculated by Eqs. (2, 3) with other parameters fixed. Figure 4c shows that a mode with low $\gamma _{exc}^r$ and high *κ*_*exc*_ is favored for the excitation enhancement, in consistency with Eq. (2). However, the emission process is more complicated and both high $\gamma _{em}^r$ and high *κ*_*em*_ are preferred for the plasmonic mode at 1050 nm (Fig. 4d). Obviously, these complicated/conflicting requirements are not easy to satisfy in symmetric nano-antennas lacking tuning parameters. The added tuning freedoms in asymmetric nano-antennas, however, offer us new opportunities to find systems supporting plasmonic modes with near-field and far-field properties better suit these requirements. Through fitting FEM calculations on realistic structures with the CMT model, we are able to retrieve the near-field (*κ*_*exe*_, *κ*_*em*_) and far-field $(\gamma _{exc}^r,\,\gamma _{em}^r)$ properties of those nano-antennas on two dashed lines in Fig. 4a, b, and then plot their evolution paths on the phase diagrams shown in Fig. 4c, d. As the structural asymmetry increases, two plasmonic modes supported by nano-antennas become “darker” exhibiting smaller $\gamma _{exc}^r\,{\mathrm{and}}\,\gamma _{em}^r$. This is not surprising since those two modes are both bright in symmetric systems, and increasing the asymmetry can bring more dark components into hybridizations, leading to decreased brightness. Meanwhile, the influences of structural asymmetry on near-field couplings (*κ*_*exe*_ and *κ*_*em*_) are more subtle but less significant, since the “local” environments inside the gap between two bars are not altered during structural deformation. As a result, enhancing structural asymmetry of nano-antennas can drive the mode at 633 nm to move into the highly favorable phase region for the excitation process (Fig. 4c), but move the mode at 1050 nm within a plateau region already exhibiting quite high *ϕ* values in the emission phase diagram (Fig. 4d). We emphasize that our experimentally fabricated nano-antennas are carefully designed to locate inside such a high-*ϕ* plateau region. An undesigned nano-antenna can easily locate at a low-*ϕ* region since its plasmonic mode does not necessarily exhibit the required NF and FF properties (κ_em_ and ${\upgamma}_{{\mathrm{em}}}^{\mathrm{r}}$). For example, red triangle in Fig. 4d represents a symmetric double-bar antenna working with its anti-binding dipole mode, yielding a low *ϕ* around 0.5. Our phase diagrams point out practical ways to design antennas with better performances. In particular, black dashed line in Fig. 4d represents a series of asymmetric nano-antennas with plasmonic modes exhibiting even higher *κ*_*em*_ (and thus higher *ϕ*), achieved by continuously reducing the gap distance *d* between two bars (see Sec. 8 in SI for more details).

**Fig. 5:**
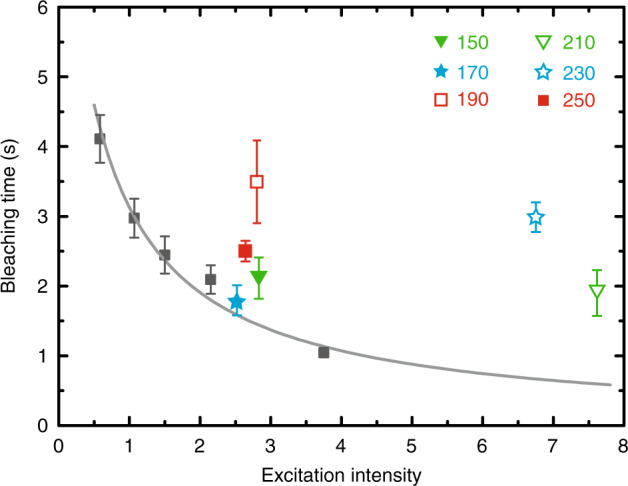
Measurement of bleaching time t_B_ as a function of b_2_. Measurement of t_B_ on glass as a function of excitation power density displays an inverse proportional relationship (gray squares and gray line). While t_B_ of molecules on antenna-array are all longer than corresponding t_B_ on glass (color symbols represent t_B_ on corresponding structure)

Finally, we demonstrate that enhancement of *ϕ* improves photostability of AIEE1000. Bleaching time *t*_*B*_, defined as the lifetime of the fluorescence molecule before being photobleached, determines the total number of photons emitted from single molecule^12,18^. As the excitation intensity gradually increases, we observe a *t*_*B*_ inversely proportional to the excitation intensity (Fig. 5; see Methods and Supplementary Information Section 9 for details), so each molecule emits roughly the same number of fluorescence photons before being photobleached. *t*_*B*_, however, significantly deviates from such trend in molecules located around asymmetric double-bar nano-antennas (Fig. 5), indicating a much higher number of fluorescence photons emitted by those molecules. The nano-antennas are, therefore, able to drastically suppress photobleaching. Because the local field enhancement does not improve photostability, the suppression comes mainly from the increased quantum yield as a result of competition between photobleaching rate and energy transfer rate to antenna.

## Discussion

We discuss three points before conclusion. First, a key advantage of our coupled double-bar structure is the additional tunable parameter—structural asymmetry. The new parameter enables us to fine-tune the near- and far-field properties of the two plasmonic modes which are already resonantly matching the excitation/emission frequencies (Fig. 4), thus reaching a more optimized fluorescence enhancement than in previous studies that lack such a degree of freedom^19^. Second, the dye molecules are fixed in PMMA matrix around antennas randomly, the limited statistics of molecules at optimized hot-spot positions causes the large discrepancy between the observed fluorescence enhancement and the theoretical limit. There is much room for further enhancing single-molecule fluorescence using asymmetric nano-antennas. Third, the optimal ranges of *F*_*exc*_ and *F*_*em*_ sometimes do not overlap in phase space. In that case, a more general guidance is to add more structurally tunable degree of freedoms to the nano-structures. By adjusting these structural parameters, one may control *F*_*exc*_ and *F*_*em*_ independently, reaching a structure yielding optimal *F*_*exc*_ and *F*_*em*_ simultaneously.

In conclusion, we design and construct plasmonic nano-antennas that significantly enhance the fluorescence signal of single AIEE1000 molecule. The double-bar nano-antennas support multiple plasmonic resonances that we engineer to simultaneously match the excitation and emission wavelengths of the molecule. More importantly, we employ the additional degree of freedom offered by structural asymmetry to fine-tune the near-field and far-field property of the plasmonic modes, reaching an optimal enhancement of ~ 405-fold. The much-increased quantum yield (from ~1% to ~80%) of AIEE1000 obtained in simulations makes the molecule potentially suitable as an NIR probe for biomedical applications. Our study establishes a novel, universal approach to enhance single-molecule fluorescence in the NIR regime.

## Materials and methods

### Sample preparation

Double-bar antennas are fabricated on 170-µm-thick transparent glass substrate (Fisher-brand, PA 15275) using electron-beam lithography (EBL, JEOL, 6300FS). We add a10-nm-thick Al layer on top of the PMMA prior to electron beam lithography to reduce beam distortion caused by surface charging. Double-bar nano-antennas are deposited with electron-beam evaporation (Kurt J. Lesker, LAB-18). The NIR fluorescent molecules AIEE1000 are mixed into PMMA gel (A2; Microchem) and spun onto the substrate at 8300 rpm to achieve a final thickness of 60 nm (Fig. 2a). Dimensions of the nano-antennas are confirmed with scanning-electron microscopy (SEM, Zeiss Sigma).

### Fluorescence observation system

Fluorescence of AIEE1000 is measured in a variable angle epifluorescence microscope (VAEM) with 633 nm laser excitation (See experimental setup in Supplementary Section 3)^20,21^. We chose appropriate excitation and emission filters to allow only fluorescence from AIEE1000 to reach the electron multiplying charge-coupled device (EMCCD, Andor-iXon-Ultra-897). Exposure time was fixed at 100 ms and gain of EMCCD was set to zero.

### Measurement of single molecule fluorescence intensity on glass without antenna

We measure the fluorescence intensity from one pixel-area changing with concentration of AIEE1000 in gradient (~2–90 molecules / pixel-area), and the slope of the intensity-concentration curve represents the single-molecule fluorescence intensity of AIEE1000 (Supplementary Fig. 5). Each molecule is randomly oriented in PMMA with respect to the elliptical excitation field polarization, and each pixel-area collects fluorescence from several to tens of molecules. In each concentration, intensities of the brightest 10 pixels are averaged as the data point on the intensity-concentration curve to calculate the brightness of unenhanced AIEE1000 with dipole moment oriented along the excitation field, **I**_**g**_, yielding 5.3 ± 0.1 detected photons per 100 ms, still over-estimated compared with the single-molecule intensity in fact.

### Numerical simulations

All FEM simulations were performed using the commercial software COMSOL Multiphysics. In studying the far-field scattering spectra (Fig. 3), we consider a 700 nm × 700 nm unit cell with periodic boundary condition imposed, containing a specific double-bar nano-antenna placed on a semi-infinite SiO_2_ substrate buried in a layer of PMMA. Permittivity of silver is given as $\varepsilon _{Ag} = \varepsilon _\infty - \frac{{\omega _p}}{{\omega (\omega + i\gamma _{Ag})}}$ with *ε*_*∞*_ = 5, *ω*_p_ = 2π × 2.175 × 10^15^ Hz, *γAg* = 2π × 4.35 × 10^12^ Hz, while permittivity of SiO_2_ is 2.28 and permittivity of PMMA is set to 1.72. The other parameters for the simulations are listed below: height of nano-bars (*h* = 50 nm), width of nano-bars (w = 50 nm), thickness of PMMA layer (h_PMMA_ = 65 nm), distance between nano-bars (*d* = 40 nm). In studying the fluorescence enhancements of our molecules (Figs. 2 and 4), we divide the whole process into two parts: excitation part and emission part. For the excitation part, we consider the same unit cell as in studying the far-field spectra, and shine the structure by normally incident plane waves. We choose a hot spot (with position fully optimized) between two bars to calculate the local electric fields with and without the nano-antenna under the illuminations of the same incident wave, and then compute the excitation enhancement based on these two fields. For the emission part, we put a dipole emitter at the same optimized hot spot as in the emission process, and then compute the total powers radiated from and absorbed by the dipole, again in the cases with and without the nano-antenna placed nearby. With these values numerically obtained in FEM simulations, we thus obtain the antenna-enabled quantum-yield enhancement of the molecule. More details can be found in Sec. 5 of Supplementary Information.

## Supplementary information

Supplementary Information for Engineering Single-molecule Fluorescence with Asymmetric Nano-antennas

## References

[1] Shashkova S, Leake MC (2017). Single-molecule fluorescence microscopy review: shedding new light on old problems. Biosci. Rep..

[2] Rhoads A, Au KF (2015). PacBio sequencing and its applications. Genomics Proteom. Bioinformatics.

[3] Moerner WE (2007). New directions in single-molecule imaging and analysis. Proc. Natl Acad. Sci. USA.

[4] Hong G, Antaris AL, Dai H (2017). Near-infrared fluorophores for biomedical imaging. Nat. Biomed. Eng..

[5] Rurack K, Spieles M (2011). Fluorescence quantum yields of a series of red and near-infrared dyes emitting at 600– 1000 nm. Anal. Chem..

[6] Tang J (2018). Selective far-field addressing of coupled quantum dots in a plasmonic nanocavity. Nat. Commun..

[7] Novotny L, van Hulst N (2011). Antennas for light. Nat. Photonics.

[8] Li J-F, Li C-Y, Aroca RF (2017). Plasmon-enhanced fluorescence spectroscopy. Chem. Soc. Rev..

[9] Yuan H, Khatua S, Zijlstra P, Yorulmaz M, Orrit M (2013). Thousand‐fold enhancement of single‐molecule fluorescence near a single gold nanorod. Angew. Chem..

[10] Kinkhabwala A (2009). Large single-molecule fluorescence enhancements produced by a bowtie nanoantenna. Nat. Photonics.

[11] Ringler M (2008). Shaping emission spectra of fluorescent molecules with single plasmonic nanoresonators. Phys. Rev. Lett..

[12] Cang H, Liu Y, Wang Y, Yin X, Zhang X (2013). Giant suppression of photobleaching for single molecule detection via the Purcell effect. Nano Lett..

[13] Qian G (2008). Band gap tunable, donor– acceptor− donor charge-transfer heteroquinoid-based chromophores: Near infrared photoluminescence and electroluminescence. Chem. Mater..

[14] Rogobete L, Kaminski F, Agio M, Sandoghdar V (2007). Design of plasmonic nanoantennae for enhancing spontaneous emission. Opt. Lett..

[15] Vandenbem C, Brayer D, Froufe-Pérez L, Carminati R (2010). Controlling the quantum yield of a dipole emitter with coupled plasmonic modes. Phys. Rev. B.

[16] Lu G (2013). Enhancing molecule fluorescence with asymmetrical plasmonic antennas. Nanoscale.

[17] Fan S, Suh W, Joannopoulos JD (2003). Temporal coupled-mode theory for the Fano resonance in optical resonators. JOSA A.

[18] Carminati R, Greffet J-J, Henkel C, Vigoureux J-M (2006). Radiative and non-radiative decay of a single molecule close to a metallic nanoparticle. Opt. Commun..

[19] Liu S-Y (2013). Simultaneous excitation and emission enhancement of fluorescence assisted by double plasmon modes of gold nanorods. J. Phys. Chem. C..

[20] Konopka CA, Bednarek SY (2008). Variable‐angle epifluorescence microscopy: a new way to look at protein dynamics in the plant cell cortex. Plant J..

[21] Sinkó J, Szabó G, Erdélyi M (2014). Ray tracing analysis of inclined illumination techniques. Opt. Express.

