# Supplementary material for: Engineering single-molecule fluorescence with asymmetric nano-antennas
Source: Light Sci Appl. 2021 Apr 14;10:79. doi: 10.1038/s41377-021-00522-9 (PMC8046762; doi:10.1038/s41377-021-00522-9)
Supplement: Supplementary file 1 — Supplementary Information for Engineering Single-molecule Fluorescence with Asymmetric Nano-antennas [file 41377_2021_522_MOESM1_ESM.docx]

*Supplementary Information for*

**Engineering** **Single-molecule Fluorescence with Asymmetric Nano-antennas**

Wenqi Zhao^1†^, Xiaochaoran Tian^1†^, Zhening Fang^1†^, Shiyi Xiao^2,3^, Meng Qiu^1^,
Qiong He^1^, Wei Feng^4^, Fuyou Li^4^, Yuanbo Zhang^1,5,6^, Lei Zhou^1,6*^, and Yan-Wen Tan^1,7*^

*^1^State Key Laboratory of Surface Physics and Department of Physics, Fudan University, Shanghai 200433, China*

*^2^Shanghai Institute for Advanced Communication and Data Science, Shanghai University, Shanghai 200444, China*

*^3^Key Laboratory of Specialty Fiber Optics and Optical Access Networks, Joint International Research Laboratory of Specialty Fiber Optics and Advanced Communication, Shanghai University, Shanghai 200444, China*

*^4^Department of Chemistry and State Key Laboratory of Molecular Engineering of Polymers, Fudan University, Shanghai 200433, China*

*^5^Institute for Nanoelectronic Devices and Quantum Computing, Fudan University, Shanghai 200433, China*

*^6^Collaborative Innovation Center of Advanced Microstructures, Nanjing 210093, China*

*^7^Multiscale Research Institute of Complex Systems, Fudan University, Shanghai 200433, China*

† These authors contributed equally to this work.

* Corresponding should be sent to: Y.-W. Tan (ywtan@fudan.edu.cn) and
L.Zhou. (phzhou@fudan.edu.cn).

**List of contents:**

**Section 1** – Quantum yield of AIEE1000 in PMMA-A2

**Section 2** – Fluorescence intensity of single AIEE1000 molecule

**Section 3** – SEM pictures of fabricated samples

**Section 4** – Structural parameters of nano-antennas and their far-field spectra

**Section 5** – Numerical evaluations of the far-field scattering spectra and the fluorescence enhancement

**Section 6** – Coupled mode theory (CMT) formalisms

**Section 7** – Retrieving CMT parameters from full-wave simulations

**Section 8** – CMT parameters of those additional structures studied in Fig. 4d

**Section 9** – Bleaching time of AIEE1000 on glass and antenna

**Supplementary References**

**Section 1 – Evaluation of Quantum yield of organic dye AIEE1000**

In order to measure the quantum yield of AIEE1000 in PMMA, IR1061 (Sigma-Aldrich) was used as the IR quantum yield standard. In both cases, the absorption peak value was below 0.2 a.u. ensuring that the re-absorption effect along the fluorescent path can be neglected^1^. The quantum yield $\Phi$ is defined as the ratio of emitted photons to the absorbed photons. Assuming an isotropic emission and a fluorescence efficiency independent of excitation wavelength, the integrated area under the emission spectrum can approximately represent the intensity of fluorescence (see Fig. S1). Refractive index of solvents should be included in calculation. Finally, the quantum yield $\phi$ can be expressed as^2^:

$\phi=\frac{Fn^{2}}{F_{\text{ref}}\cdot n_{\text{ref}}^{2}}\cdot\frac{I_{0}^{\text{ref}}[1-\exp(-A_{ref})]}{I_{0}[1-\exp(-A)]}\cdot\phi_{ref}$ (S1)

Where $F$ is the integrated fluorescence intensity over the whole spectrum, $n$ and $n_{ref}$ are the refractive indexes of solvents for sample and standard. $Q_{a}=I_{0}[1-exp\{-A\}]$ represents the absorbed photons, with $I_{0}$ is the number of incident photons and $A$ is the absorbance of solution. $\phi_{ref}$ is the quantum yield of IR1061 in Dichloromethane methylene chloride (DCM) at room temperature. Parameters used in calculation and $\phi$ of AIEE1000 are shown in Table S1. $\phi$ of AIEE1000 in PMMA-A2 is 1.19 %, much smaller than in toluene ($\phi=7.1\%$) and in 90% methanol-THF (2.9%)^3^.

**Table S1**：parameters of AIEE1000 and IR1061. Here, F, A and n denote integrated fluorescence intensity over the whole spectrum, absorbance of solution, and refractive indexes, respectively.

|  | ***F*** | ***A*@730nm** | ***n* of solvents** | $\phi$ |
| --- | --- | --- | --- | --- |
| **IR1061** | 8.15 | 0.025 | 1.42 | 0.017 |
| **AIEE1000** | 12.15 | 0.060 | 1.49 | 0.0119 |


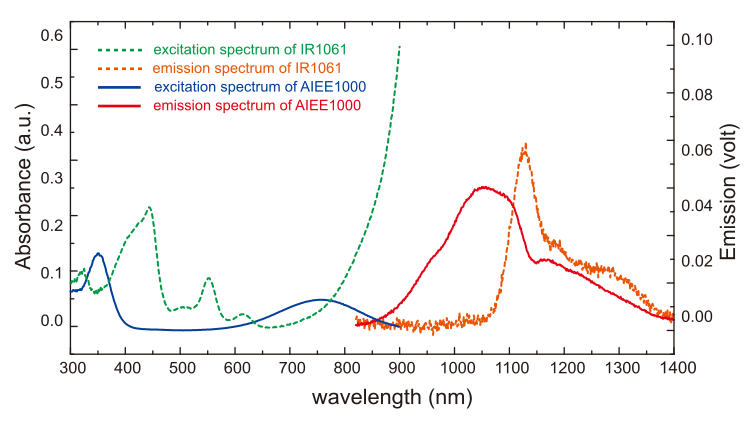


**Figure S1：**Absorption spectra of 10uM solutions of: (dashed-green line) IR1061 in DCM; (solid-blue line) AIEE1000 in PMMA-A2; Emission spectra of same solutions of: (dashed-orange line) IR1061 in DCM; (dashed-red line) AIEE1000 in PMMA-A2.

**Section 2. Fluorescence intensity of single AIEE1000 molecule**

To measure the fluorescence intensity of single AIEE1000 molecule without antenna, we increase the concentration of AIEE1000 in gradient and disperse molecules into PMMA layer (80nm) randomly. Experimental setup shown in Fig. S2 is used to excite AIEE1000 and collect the fluorescence emission. Assuming that single-molecule fluorescence intensity is $I_{0}$, at concentration $C_{1}$, fluorescence intensity from the molecules dispersed in single pixel volume is determined by $I_{1}=I_{0}C_{1}A_{pixel}h$ , with $A_{pixel}$ being area of single pixel and *h* being the height of PMMA-layer. For different concentration $C_{n}$, single-pixel volume fluorescence intensity changes as $I_{n}=I_{0}C_{n}A_{pixel}h$. Fig. S3 illustrate the evaluation of single pixel volume intensity verses concentration. The slope of linear fitting represents the single molecule fluorescence intensity in experimental condition, indicating that he $I_{0}=5.304$ counts per frame (cpf).


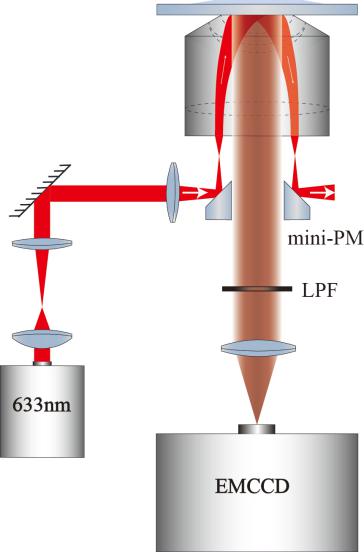


**Figure S2：**Schematics of home-built microscopic single-molecule fluorescence detection system, which consist of a 633nm excitation module, a microscope and a fluorescence collection module. LPF, 850 nm long-pass filter; EMCCD, electron multiplying charge coupled device; mini-PM, mini-plane mirror, tilted at the outer-ring of objective.


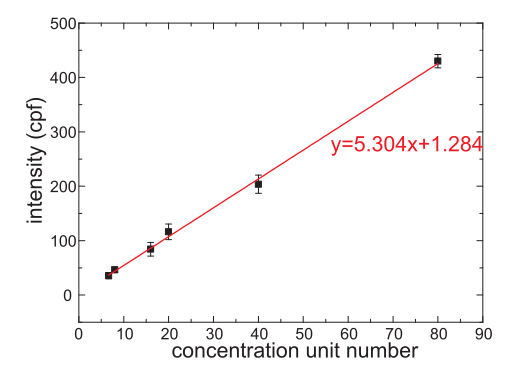


**Figure S3:** Relationship between fluorescence intensity and AIEE1000’s concentration. Black-squares are the averages of statistically counted fluorescence intensities from one pixel-area, exposure-time and power density were set at 100ms and 32 kW/cm^2^ respectively. The error-bars come from the standard deviations of fluorescence intensities.

**Section 3– SEM pictures of fabricated samples**


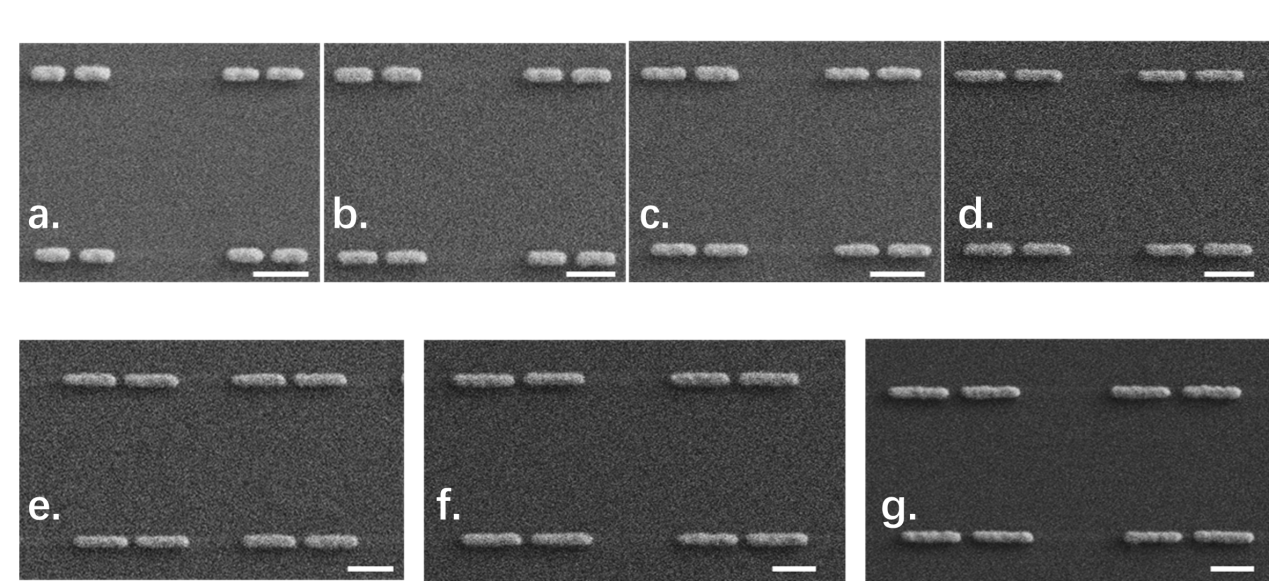


**Figure S4：**Scanning electron microscope (SEM) pictures of fabricated samples containing periodic arrays of symmetric double-bar antennas. The lengths of the nano-bars are **a.** 135 nm, **b.** 155 nm, **c.** 180 nm, **d.** 200 nm, **e.** 230 nm, **f.** 265 nm, and **g.** 280 nm, respectively. Scale: 200 nm.


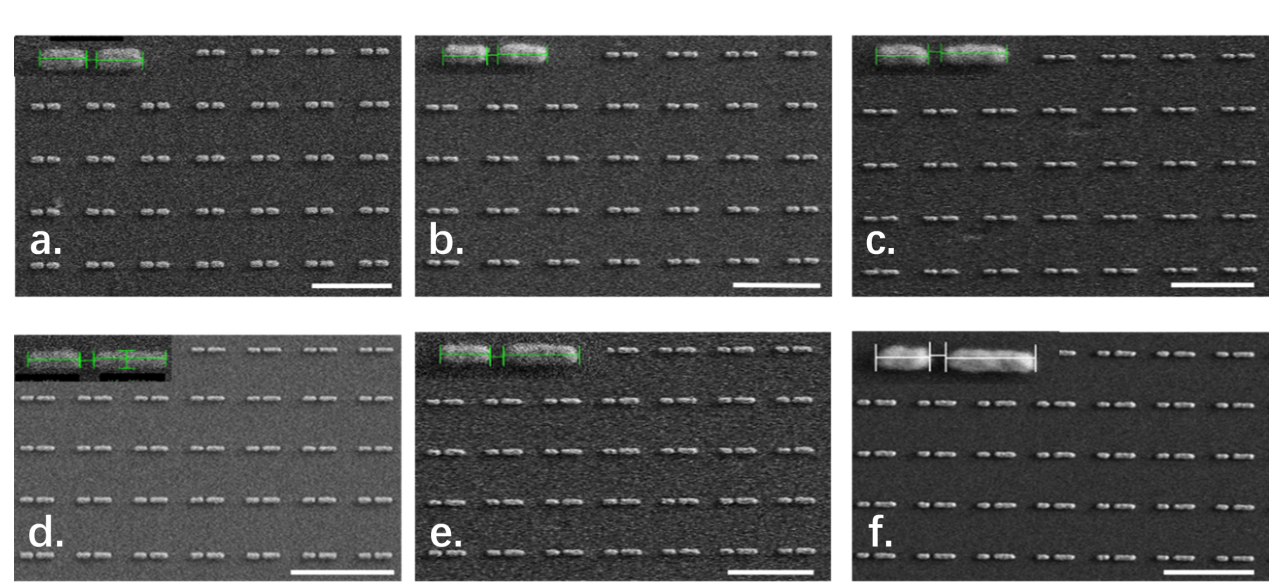


**Figure S5：**Scanning electron microscope (SEM) pictures of fabricated samples containing periodic arrays of asymmetric double-bar antennas. The lengths of the shorter nano-bars are fixed at 150 nm, while that of the longer ones are **a.** 150 nm, **b.** 170 nm, **c.** 190 nm, **d.** 210 nm, **e.** 230 nm, and **f.** 250 nm, respectively. Scale: 1 um.

**Section 4** **– Structural parameters of nano-antennas and their far-field spectra**

The structural parameters (see inset of Fig. S6a for the definitions of parameters) of the antennas used in experimentally reported nano-system are estimated as follows. First of all, the widths and rough lengths (shown in Table S2-S3) are estimated according to SEM results (Fig. S4-S5). We acquire the optical responses of antennas through illuminating array-area vertically and collecting the transmitted signal, comparing with signal from substrate-area, we get the transmission spectrum specifically (see Fig. S6).

**Table S2:** structure parameters according to SEM results of asymmetric double-bar antennas

| Double-bar antenna | $\boldsymbol{b}_{\boldsymbol{1}}$ (nm) | $\boldsymbol{b}_{\boldsymbol{2}}$ (nm) | Gap (nm) |
| --- | --- | --- | --- |
| 150 | 151.48±2.67 | 150.51±1.89 | 34.91±4.05 |
| 170 | 150.55±0.93 | 170.76±2.86 | 34.35±4.54 |
| 190 | 151.12±0.73 | 190.62±3.45 | 34.37±4.55 |
| 210 | 152.44±2.04 | 210.94±3.99 | 34.86±4.16 |
| 230 | 152.08±2.44 | 231.01±1.56 | 33.62±2.31 |
| 250 | 151.71±2.63 | 248.16±2.86 | 33.70±2.18 |

**Table S3:** structure parameters according to SEM results of symmetric double-bar antennas

| Double-bar antenna | $\boldsymbol{b}_{\boldsymbol{1}}$ (nm) | $\boldsymbol{b}_{\boldsymbol{2}}$ (nm) | Gap (nm) |
| --- | --- | --- | --- |
| 135 | 134.57±4.18 | 136.86±3.65 | 32.07±7.21 |
| 150 | 152.44±5.11 | 153.53±4.42 | 39.94±4.62 |
| 180 | 178.01±5.21 | 178.95±3.23 | 39.82±5.47 |
| 200 | 197.45±4.35 | 199.50±3.48 | 43.38±4.32 |
| 230 | 230.29±3.82 | 233.23±2.99 | 40.56±6.22 |
| 260 | 259.62±5.22 | 261.17±5.41 | 45.78±4.78 |
| 280 | 274.30±8.90 | 276.42±8.45 | 68.86±9.26 |


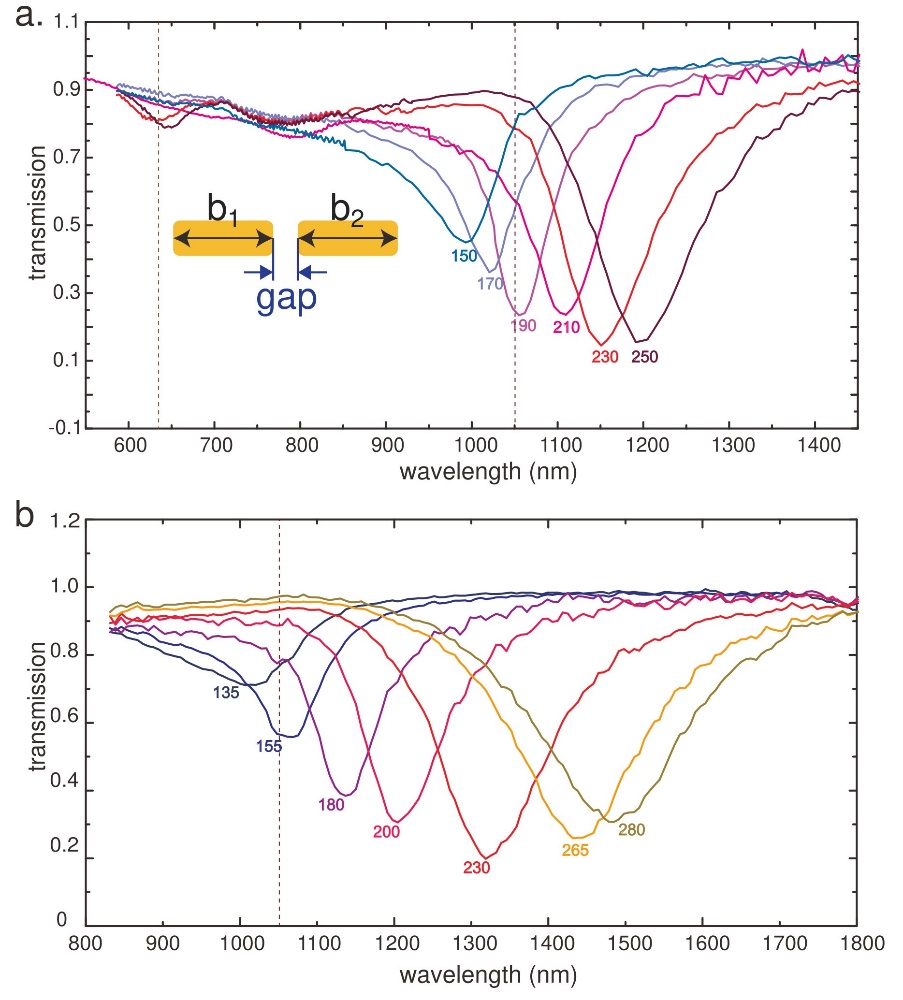


**Figure S6：**Transmission spectra of DB-antennas. **a.** Asymmetric structures with $b_{1}=150nm$, $b_{2}$ changes from 150 nm to 250 nm, respectively. **b.** Symmetric structures with $b_{1}=b_{2}$ changes from 135 nm to 280 nm, respectively. Yellow and purple dotted-line stands for excitation and emission wavelength in fluorescence experiments (633 nm and 1050 nm). Inset: Definition of DB-antenna parameters.

**Section 5 – Numerical evaluations of the far-field scattering spectra and the fluorescence enhancement**

Fluorescence enhancement rate of a single molecule is a product of the enhancement rates in its excitation and quantum yield. Therefore, fluorescence enhancement can be expressed as

|  | $F_{E}=F_{exc}\cdot F_{em}$ | (S2) |
| --- | --- | --- |

where $F_{exc}\equiv G_{exc}/G_{exc}^{0}$ and $F_{em}\equiv\phi/\phi^{0}$, with $G_{exc}$ and $\phi$ denoting the excitation rate and quantum yield of the molecule with a nano-antenna placed nearby, while those with superscript “0” denote the free-space counterparts. .
**5.1. Numerical evaluations of** $\boldsymbol{F}_{\boldsymbol{exc}}$ **and the far-field scattering spectra**

The excitation rate of a molecule $G_{exc}$ is proportional to

|  | $G_{exc}\sim\left\vert\boldsymbol{E}_{\boldsymbol{local}}\boldsymbol{\cdot}\boldsymbol{p}_{exc} \right\vert^{2}$ |  |  |  | (S3) |
| --- | --- | --- | --- | --- | --- |

where $\boldsymbol{E}_{\boldsymbol{local}}$ denotes the local electric field experienced by the molecule and $\boldsymbol{p}_{exc}$ is the molecule’s transition dipole moment at the excitation frequency. As an intrinsic property of a molecule, $\boldsymbol{p}_{exc}$ is essentially a constant independent of the presence of a nano-antenna, and hence the enhancement of excitation rate $F_{exc}$ is

|  | $F_{exc}=\frac{G_{exc}}{G_{exc}^{0}}=\frac{\left\vert\boldsymbol{E}_{\boldsymbol{local}} \right\vert^{2}}{\left\vert\boldsymbol{E}_{\boldsymbol{0}} \right\vert^{2}},$ | (S4) |
| --- | --- | --- |

where $\boldsymbol{E}_{\boldsymbol{0}}$ is the electric field felt by the molecule without a nano-antenna placed nearly, under the same external illumination.

We now describe how to numerically evaluate $F_{exc}$ based on Eq. (S4). In our finite-element-method (FEM) simulations, we study a $700 nm \times700 nm$ unit cell containing a nano-antenna with periodic boundary conditions imposed, and shine the system by normally incident plane waves (see Fig. S7). Since the local electric field inside the gap is largely parallel to the bar (denoted as the *x* direction), we use $F_{exc}={\left| E_{\boldsymbol{local,x}} \right|^{2}}/{\left| E_{\boldsymbol{0,x}} \right|^{2}}$ to evaluate the excitation enhancement, after we numerically obtain the local electric field distributions with or without the nano-antenna, under the same illumination. To better mimic the realistic situations, we simulate the averaged electric field $\boldsymbol{E}_{\boldsymbol{local}}$ in an optimized hot-spot region (within an area of $2\times2\times2 nm$, see the red spot in Fig. S7) inside the gap between two nano bars. Note that this hot-spot position is fully optimized in our simulations based on the condition that it yields the maximized $F_{E}$ not just $F_{exc}$.

The far-field scattering (including transmission *T*, reflection *R* and absorption *A*) spectra of the nano-antenna arrays can be computed by FEM using exactly the same simulation setup as described above.


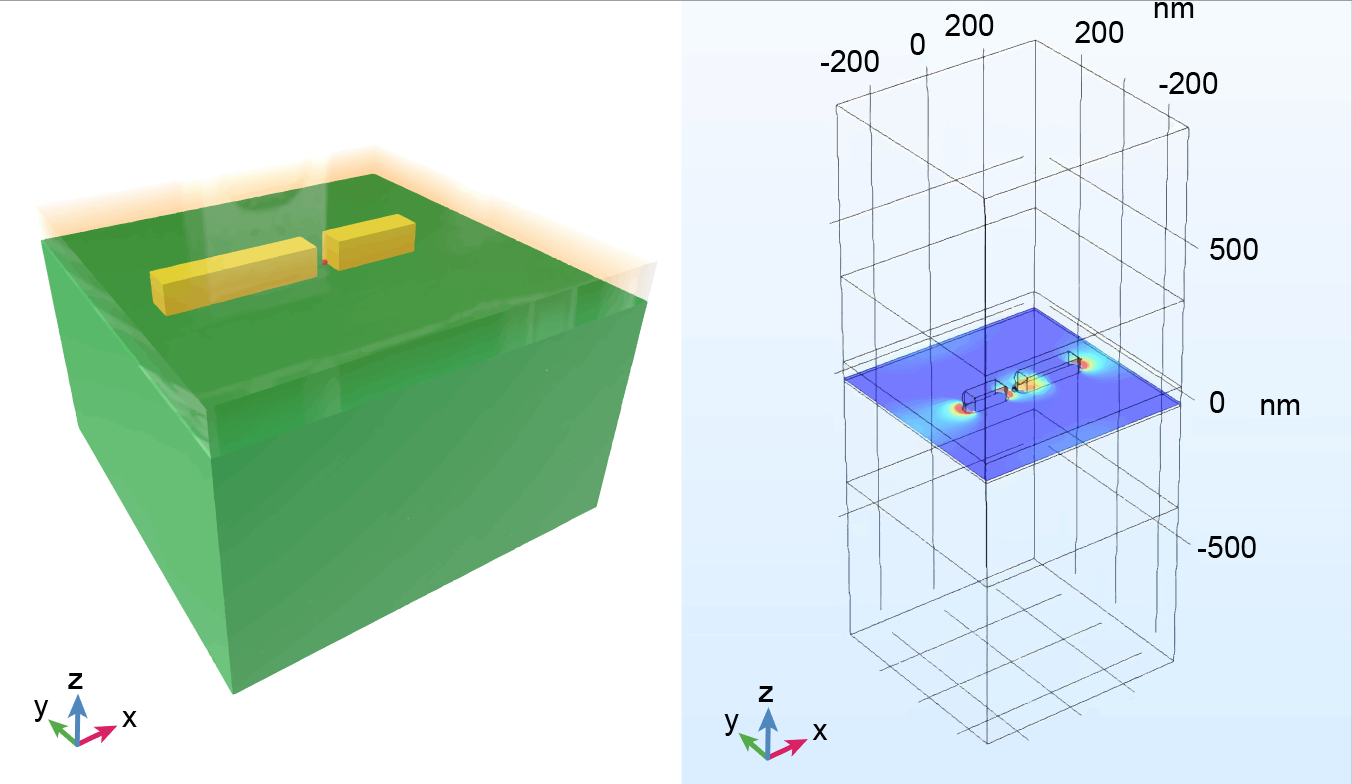


**Figure S7.** Simulation schematics for computing the excitation enhancement. In our simulations, we used $\mathrm{SiO}_{2}$ as the dielectric substrate, and all nano-systems are embedded in PMMA.

**5.2. Numerical evaluations of** $\boldsymbol{\phi}$ **and** $\boldsymbol{F}_{\boldsymbol{em}}$

The intrinsic quantum yield of a molecule is defined as

|  | $\phi^{0}=\frac{\gamma_{0}^{r}}{\gamma_{0}^{r}+\gamma_{0}^{nr}}$ | (S5) |
| --- | --- | --- |

where $\gamma_{0}^{r}$ and $\gamma_{0}^{\mathrm{nr}}$ denote, respectively, the radiative and nonradiative decay rates of the molecule placed in vacuum. Meanwhile, quantum yield of the molecule, with a nano-antenna placed nearby, is given by

|  | $\phi=\frac{\Gamma_{em}^{r}}{\Gamma_{em}^{r}+\Gamma_{em}^{nr}},$ | (S6) |
| --- | --- | --- |

where $\Gamma_{em}^{r}$ and $\Gamma_{em}^{nr}$ denote the radiative and nonradiative decay rates of the whole molecule+antenna system, respectively. The total nonradiative decay rate of the molecule+antenna system can be re-written as

$\Gamma_{em}^{nr}=\Gamma_{em}^{i}+\gamma_{em}^{nr}$, (S7)

which contains the absorptions contributed by the nano-antenna ($\Gamma_{em}^{i}$) and the molecule ($\gamma_{em}^{nr})$. Put Eq. (S7) to Eq. (S6) and using the definition of $\phi^{0}$ (Eq. (S5)), we can re-write Eq. (S6) as

|  | $\phi=\frac{\Gamma_{em}^{r}/\gamma_{0}^{r}}{\Gamma_{em}^{r}/\gamma_{0}^{r}+\Gamma_{em}^{i}/\gamma_{0}^{r}+(1-\phi^{0})/\phi^{0}}.$ | (S8) |
| --- | --- | --- |

Here, we have assumed that the antenna does not influence the intrinsic nonradiative decay rate of the molecule (i.e., $\gamma_{em}^{nr}=\gamma_{0}^{nr}$).

To numerically compute $\phi$, we employ FEM simulations to calculate the total energy flux radiated from a classic dipole (with a moment $\boldsymbol{p}_{em}$**)** placed in the gap between two nano-bars, using

|  | $P_{r}=\int_{s} \boldsymbol{S}\cdot d\boldsymbol{A},$ | (S9) |
| --- | --- | --- |

where $\boldsymbol{S}$ denotes the Poynting vector on a sphere with a large-enough radius surrounding the dipole (see Fig. S8a). Here, the classic dipole^4^ is located at exactly the same hot spot as in our simulations on computing the excitation rate (see Fig. S7). We emphasize once again that this hot-spot position is fully optimized in our simulations based on the condition that it yields the maximized $F_{E}$ not just $F_{exe}$ or $F_{em}$. Based on the same simulation scheme but with the double-bar antenna taken away, we numerically calculate the total energy flow radiated from the dipole without the nano-antenna (see Fig. S8b), using


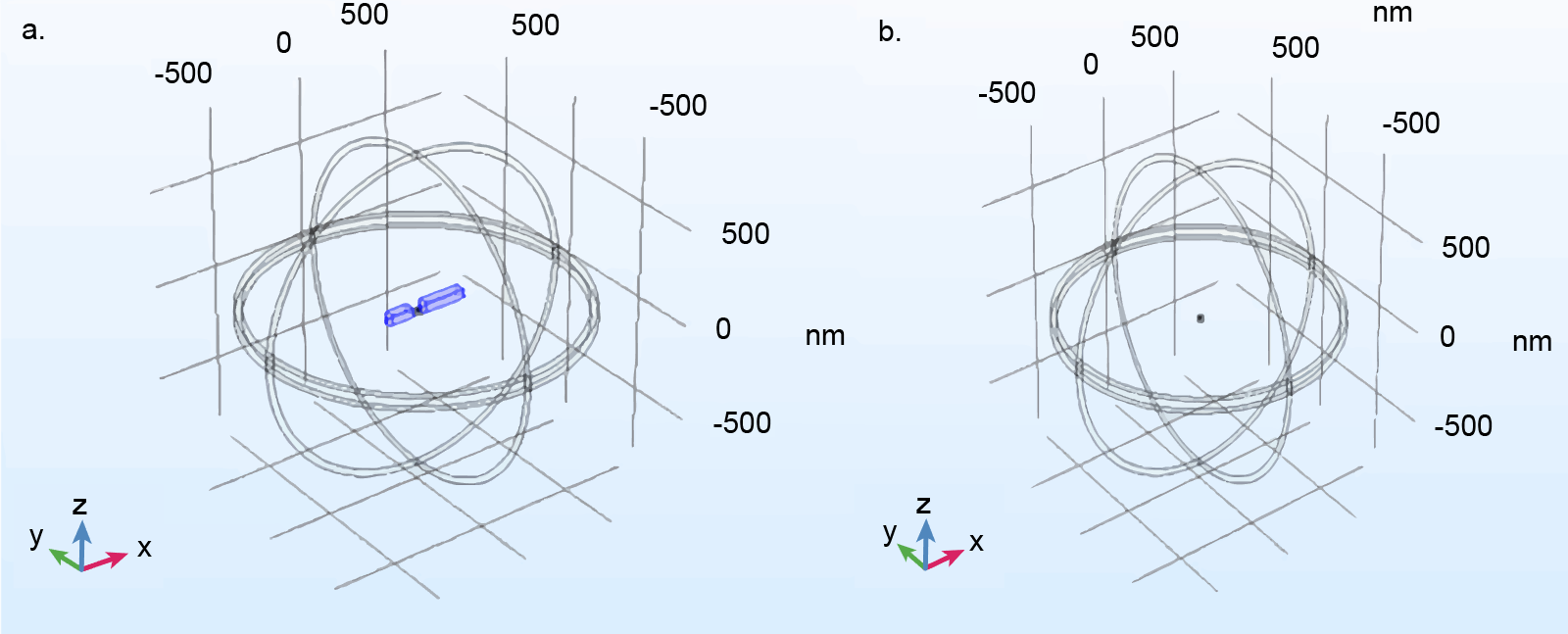


**Figure S8.** Simulation schematics for computing the quantum yield of a molecule **a.** with a nano-antenna, and **b.** without a nano-antenna.

|  | $P_{r}^{0}=\int_{s} \boldsymbol{S}^{\boldsymbol{0}}\cdot d\boldsymbol{A}$. | (S10) |
| --- | --- | --- |

We next calculate the absorption rate of our nano-antenna using

|  | $P_{i}=\int_{V} Q_{e}d\tau$, | (S11) |
| --- | --- | --- |

where $Q_{e}$ stands for the local ohmic-loss rate of the nano-antenna. Based on Poynting’s theorem, we can obtain the radiation decay rate of the molecule ($\gamma_{0}^{r})$and the radiation/absorption decay rates of the molecule-antenna system ($\Gamma_{em}^{r},\Gamma_{em}^{i}$) via

|  | $\left\{ \begin{aligned} \gamma_{0}^{r}\propto\frac{P_{r}^{0}}{2\left\vert\boldsymbol{p}_{em} \right\vert^{2}} \\ \Gamma_{em}^{r}\propto\frac{P_{r}}{2\left\vert\boldsymbol{p}_{em} \right\vert^{2}} \\ \Gamma_{em}^{i}\propto\frac{P_{i}}{2\left\vert\boldsymbol{p}_{em} \right\vert^{2}} \end{aligned} \right.$ | (S12) |
| --- | --- | --- |

up to a common factor. Instead of determining the proportional factor in Eq. (S12), we are more interested in the following ratios:

|  | $\left\{ \begin{matrix} \frac{\Gamma_{em}^{r}}{\gamma_{0}^{r}}=\frac{P_{r}}{P_{r}^{0}} \\ \frac{\Gamma_{em}^{i}}{\gamma_{0}^{r}}=\frac{P_{i}}{P_{r}^{0}} \end{matrix} \right.,$ | (S13) |
| --- | --- | --- |

which can be unambiguously determined once$P_{r}{,P}_{r}^{0}{and P}_{i}$ are numerically evaluated using Eqs. (S10-S12). Substituting Eq. (S13) into Eq. (S8), we finally obtain the quantum yield $\phi$ based on full-wave simulations. Note that$\phi^{0}$ is an intrinsic property of the molecule and is known at the very beginning, we thus finally obtain $F_{\mathrm{em}}$ using $F_{\mathrm{em}}=\phi/\phi^{0}$.

**Section 6 – Coupled-mode-theory (CMT) formalisms**

**6.1 CMT model for the excitation process**

We develop a CMT model to describe the excitation process. For simplicity, we assume that only one plasmonic mode of the nano-antenna is important at frequencies around the excitation frequency. As shown in Fig. S9a, consider that a light beam is normally incident on the nano-antenna array, we have the following CMT equations

|  | $\frac{\partial}{\partial t}a_{exc}=\left( -i\omega_{exc}-\gamma_{exc}^{r}-\gamma_{exc}^{i} \right)a_{exc}+d_{exc}^{0}S_{exc,0}^{+},$  $S_{exc,n}^{-}=C_{exc}^{n0}S_{exc,0}^{+}+d_{exc}^{n}a_{exc},$ | (S14) |
| --- | --- | --- |

to describe the dynamics of the plasmonic mode and the total wave scatterings to the far-field ports. Here, $a_{exc}$ denotes the (complex) amplitude of the excited plasmonic mode with resonant frequency $\omega_{exc}$ and radiation/absorption damping rates$\gamma_{exc}^{r}$ and $\gamma_{exc}^{i}$, $S_{exc,0}^{+}$ represents the strength of incident light and $S_{exc,n}^{-}$describes the strength of scattered light to the *n*-th port, $d_{exc}^{n}$ describes the coupling strength between the plasmonic mode and the *n*-th external port, and $C_{exc}^{n0}$ denotes the scattering coefficient to the *n*-th channel of the background system (with all antennas taken away, see Fig. S9b). In our experiments/simulations, the system under study contains a periodic array of nano-antennas with lattice constant 700 nm larger than the wavelength of light (633 nm), and thus light diffractions inevitably exist. The 0^th^ and 1^st^ channels label the normal reflection and transmission channels, while those with *n* > 1 describe other diffraction channels (see Fig. S9a). According to this definition, we find that only two elements in matrix $C_{exc}^{n0}$ are non-zero, which are $C_{exc}^{00}=r_{0} \mathrm{and} C_{exc}^{10}=t_{0}$ describing the reflection/transmission coefficients of the background (i.e., the substrate), obtainable from the numerical calculations for the background system (see Fig. S9b).


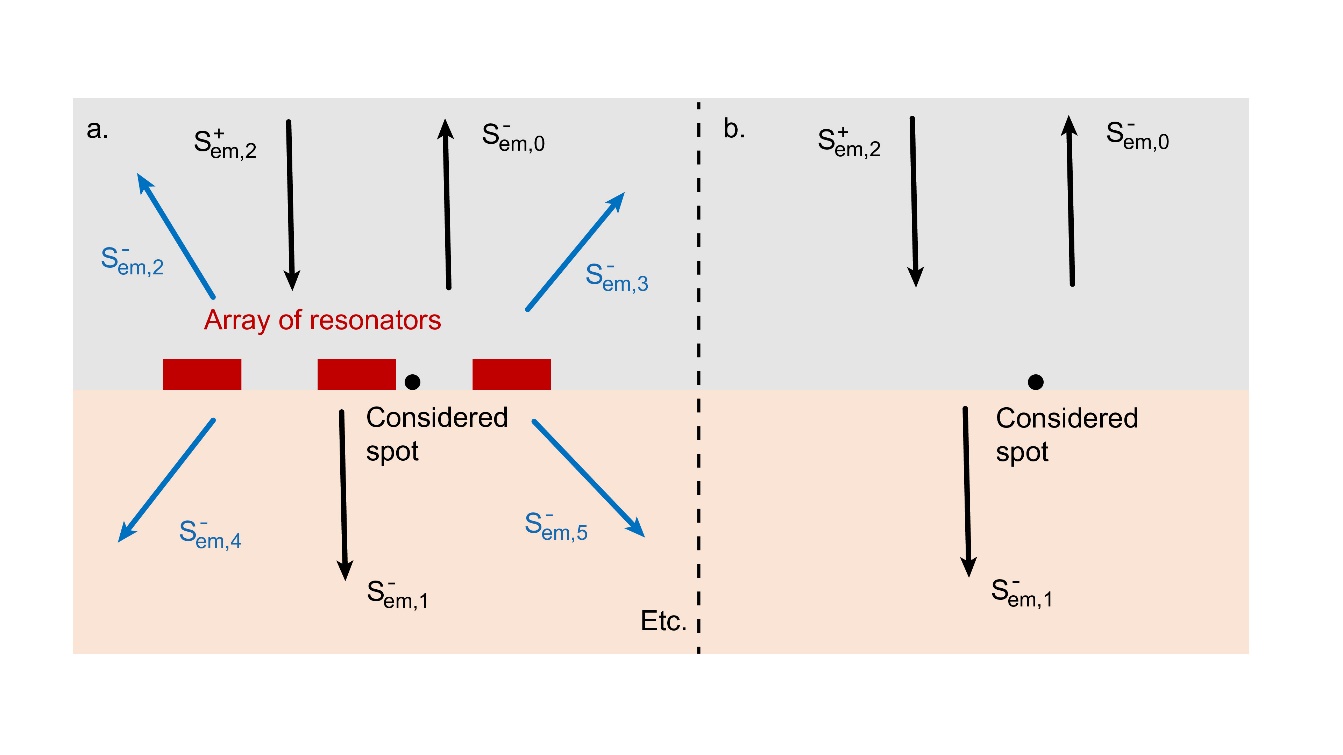


**Figure S9**. A sketch of the CMT model for the excitation process. **a.** The nano-antenna array is shined by a normally incident plane wave coming from the 0^th^ port. Normal reflection and transmission channels are denoted as the 0^th^ and 1^st^ outgoing ports, respectively. Other ports denote high-order diffraction channels. **b.** Shine the same system but with all nano-antennas removed to obtain the local electric fields inside the same region.

Solving the first line in Eq. (S14), we get

$a_{exc}=\frac{d_{exc}^{0}S_{exc,0}^{+}}{-i\left( \omega-\omega_{exc} \right)+\gamma_{exc}^{r}+\gamma_{exc}^{i}}$ . (S15)

The total field inside the system contains two parts: 1) the total field (including the incident and scattered ones) due to the background and 2) the radiated field due to the excited mode. Obviously, the second part is much stronger than the first one at frequencies near resonance, so that we only consider this part. Further, we assume that the *normalized* wave-function of the plasmonic mode is given by $\boldsymbol{E}_{mode}(\boldsymbol{r})$ and then define a parameter $\kappa_{exc}=\left\langle\boldsymbol{r}_{0} | \boldsymbol{E}_{mode} \right\rangle$ to characterize the coupling between the molecule placed at $\boldsymbol{r}_{0}$and the plasmonic mode. Based on these definitions, we finally get the following formula

| ${\vert E_{local}\vert}^{2}=\left\vert a_{exc}*\kappa_{exc} \right\vert^{2}=\left\vert\frac{\kappa_{exc}d_{exc}^{0}S_{exc,0}^{+}}{-i\left( \omega-\omega_{exc} \right)+\gamma_{exc}^{r}+\gamma_{exc}^{i}} \right\vert^{2}= \frac{\left\vert\kappa_{exc}d_{exc}^{0}S_{exc,0}^{+} \right\vert^{2}}{\left( \omega-\omega_{exc} \right)^{2}+\left( \gamma_{exc}^{r}+\gamma_{exc}^{i} \right)^{2}},$ | (S16) |
| --- | --- |

to model the realistic local field measured at the point $\mathbf{r}_{0}$where a molecule is placed.

Removing all nano-antennas, we use FEM simulations to re-calculate light scatterings at the background medium (see Fig. S9b) to get the local field measured at the same point but without the antenna. We find that

$E_{0}=C\times S_{exc,0}^{+}$ (S17)

where $C=1+r_{0}$ is nearly a constant that can be computed via FEM simulations (see Fig. S9b). Thus, we finally get

| ${F_{exc}=\vert\frac{E_{local}}{E_{\boldsymbol{0}}}\vert}^{2}=\frac{1}{{\vert C\vert}^{2}}\frac{\left\vert\kappa_{exc} \right\vert^{2}\left\vert d_{exc}^{0} \right\vert^{2}}{{\left( \omega-\omega_{exc} \right)^{2}+\left( \gamma_{exc}^{r}+\gamma_{exc}^{i} \right)}^{2}}=\frac{\left\vert d_{exc}^{0} \right\vert^{2}}{{\vert C\vert}^{2}}\frac{\left\vert\kappa_{exc} \right\vert^{2}}{\left( \gamma_{exc}^{r}+\gamma_{exc}^{i} \right)^{2}}$ | (S18) |
| --- | --- |

with the last equation obtained at the resonance frequency $\omega=\omega_{exc}$. We thus derived out Eq. (2) used in the main text.

We now derive CMT formulas for the reflection/absorption spectra of the nano-antenna array, which can be used to fit with the FEM-calculated spectra. Solving the second-line equation in Eq. (S16) with *n*=0 and consider the absorption rate of the system, we obtain that

$R=\frac{{|S_{exc,0}^{-}|}^{2}}{{|S_{exc,0}^{+}|}^{2}}={|d_{exc}^{0}\frac{d_{exc}^{0}}{-i\left( \omega-\omega_{exc} \right)+\gamma_{exc}^{r}+\gamma_{exc}^{i}}|}^{2}=\frac{\left| d_{exc}^{0} \right|^{4}}{\left( \omega-\omega_{exc} \right)^{2}+\left( \gamma_{exc}^{r}+\gamma_{exc}^{i} \right)^{2}},$ (S19)

$A=\frac{P_{nr}^{ex}}{{|S_{exc,0}^{+}|}^{2}}=\frac{{2\gamma_{exc}^{i}|a_{exc}|}^{2}}{{|S_{exc,0}^{+}|}^{2}}=2\gamma_{exc}^{i}\frac{\left| d_{exc}^{0} \right|^{2}}{\left( \omega-\omega_{exc} \right)^{2}+\left( \gamma_{exc}^{r}+\gamma_{exc}^{i} \right)^{2}}.$ (S20)

In practice, usually we first numerically obtain the spectra of$F_{\mathrm{exc}}$, $R$ and $A$ using the calculation methodology described in Sec. 5, and then use Eqs. (S18-S20) to fit these spectra to retrieve the needed CMT parameters $\kappa_{exc,} d_{exc}^{0},\gamma_{exc}^{r} \mathrm{and}\gamma_{exc}^{i}$.

We discuss the physics of these CMT parameters. Note that time-reversal symmetry and energy conservation tell us that$\gamma_{exc}^{r}=(1/2)\sum_{n} {|d_{exc}^{n}|}^{2}$. Here, due to the presence of high order diffractions, $d_{exc}^{0} \mathrm{and}\sqrt{{2\gamma}_{exc}^{r}}$ are *not* identical as in the standard one-mode two-port model, and therefore, we treat them as two independent model parameters in our fitting processes. However, they are correlated with each other, as they both describe the mode’s radiative capability to the far field, with one ($d_{exc}^{0})$ describing that to a particular channel and another ${(\gamma}_{exc}^{r})$ to all channels. Meanwhile, $\kappa_{exc}$ is obviously a different parameter determined by the near-field property of the mode and the spot $\boldsymbol{r}_{0}$where the molecule is placed.

In order to demonstrate mode variations more deeply, we illustrate in Fig. S10a the dependence of $\kappa_{exc}$ and $\gamma_{exc}^{r}$ as the structural asymmetry increases along the blue dashed line in Fig. 4c in the main text. We see that when the degree of asymmetry is enlarged, the plasmonic mode supported by nano-antennas becomes “darker” exhibiting smaller $\gamma_{exc}^{r} \mathrm{and} \gamma_{em}^{r}$ (indicated by red lines), this is not surprising since those two modes are both bright in symmetric systems, and increasing the asymmetry can bring more dark components into hybridizations, leading to decreased brightness. Meanwhile, the influences of structural asymmetry on near-field couplings ($\kappa_{exc})$are more subtle but less significant (indicated by less variations of blue lines), since the “local” environments inside the gap between two bars are not strongly altered during structural deformation. Such a structural change leads to the strengthen of local field, resulting in large enhancement in the excitation process, in agreement with our calculation.


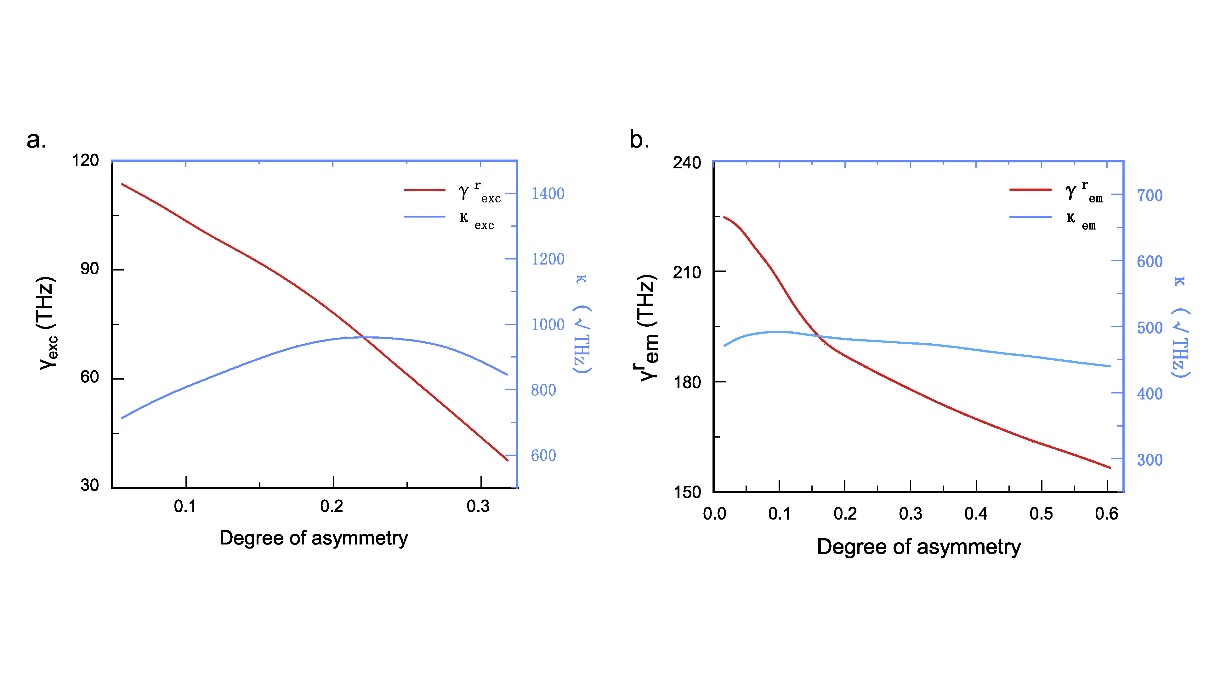


**Figure S10:** detailed analyses of near-field and far-field couplings. $\kappa_{exc}$ (blue line) and $\gamma_{exc}^{r}$ (red line) as function of degree of asymmetry for **a.** excitation (at 633 nm) and **b.** emission (at 1050 nm). Degree of asymmetry is defined as $(b_{2}-b_{1})/(b_{2}+b_{1} )$.

- 1. **CMT model for the emission process**

We now establish a CMT model for the emission process, as schematically depicted in Fig. S11. To capture the essential physics only, we make the following three assumptions: 1) the emitting molecule is an energy source which only provides energy to the nano-antenna but is not back-affected by the nano-antenna; 2) only a single plasmonic mode of the nano-antenna is relevant to the emission process; 3) only a single nano-antenna (rather than an antenna array as in excitation process) is considered here since the molecule can only efficiently couple to one resonator inside the array.


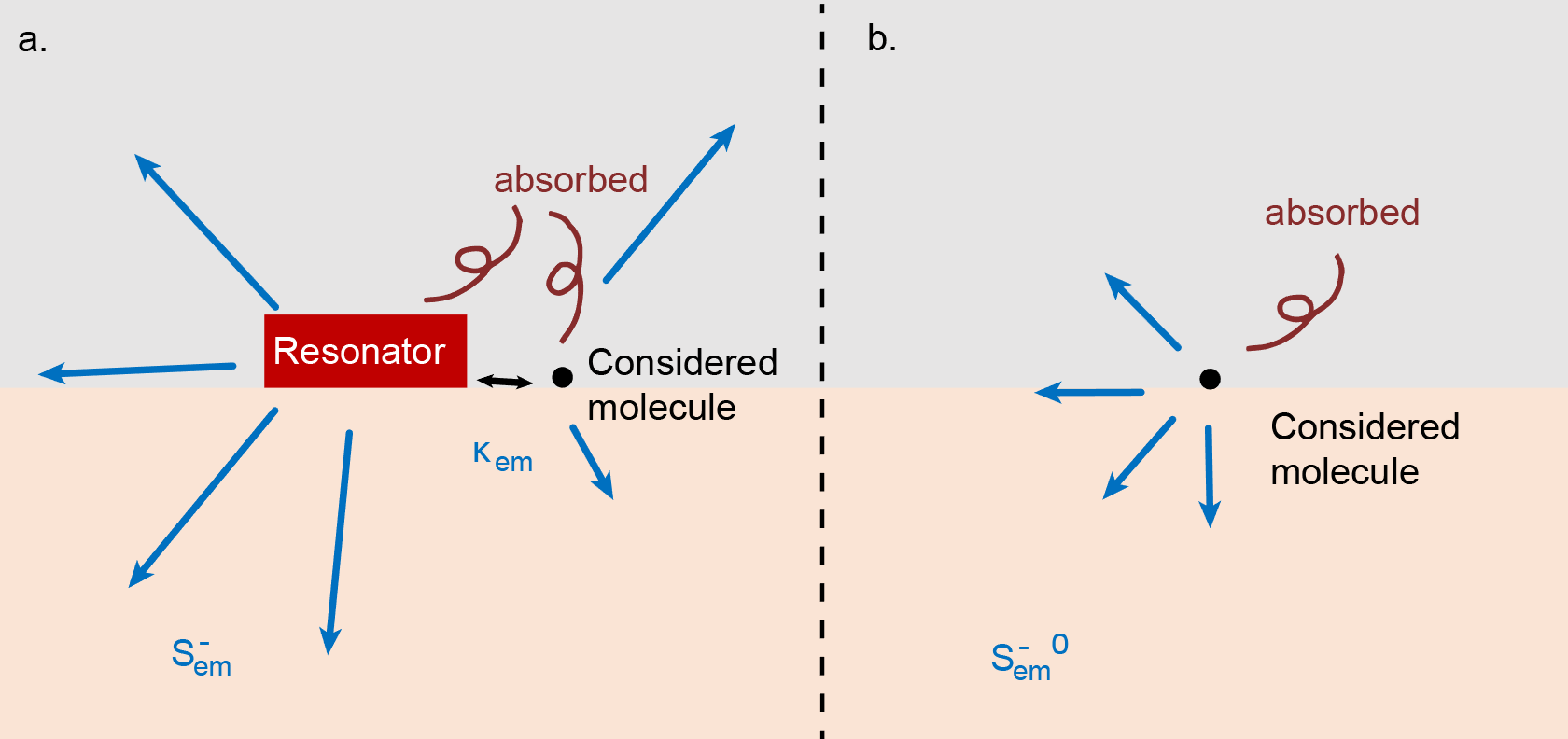


**Figure S11**. A sketch of the CMT model describing the emission process of a molecule **a.** with a nano-antenna placed nearby, and **b.** without a nano-antenna placed nearby.

Based on the above approximations, we obtain the following CMT equations:

|  | $\frac{\partial}{\partial t}a_{mode}=\left( -i\omega_{0}-\gamma_{em}^{r}-\gamma_{em}^{i} \right)a_{mode}+\kappa_{em}a_{em},$  $S_{em}^{-}=\sqrt{2\gamma_{0}^{r}}a_{em}+\sqrt{2\gamma_{em}^{r}}a_{mode},$ | (S21) |
| --- | --- | --- |

describing the mode dynamics and the radiations of the molecule + antenna to the far field, respectively. Here, $a_{mode}$ denotes the (complex) amplitude of plasmonic mode of the nano-antenna with resonance frequency $\omega_{0}$, radiation damping $\gamma_{em}^{r}$ and absorption damping $\gamma_{em}^{i}$, while $\kappa_{em}$ is the coupling strength between the emitting molecule and the nano-antenna, $S_{em}^{-}$ stands for the strength of radiation field measured at the far field port, and $a_{em}$ denotes the amplitude of the mode supported by the emitting molecule with $\gamma_{0}^{r}$ describing its radiation damping rate. In the zero-order approximation, both the emitting molecule and the nano-antenna can be effectively viewed as electric dipoles (with different amplitudes) seen at the far-field. Therefore, only one external port (the radiation channel for a dipole) is retained in our CMT model.

Solving Eq. (S20), we obtain

|  | $P_{r}={\vert S_{em}^{-}\vert}^{2}=2\gamma_{0}^{r}\left\vert a_{em} \right\vert^{2}+2\gamma_{em}^{r}\frac{\left\vert\kappa_{em} \right\vert^{2}\left\vert a_{em} \right\vert^{2}}{\left( \omega-\omega_{0} \right)^{2}+\left( \gamma_{em}^{r}+\gamma_{em}^{i} \right)^{2}},$ | (S22) |
| --- | --- | --- |

to describe the total radiation power of the molecule + antenna system. Note that obviously the two terms describe, respectively, the contributions from the molecule itself and the nano-antenna excited by the molecule.

We now calculate the absorption rate of the antenna due to plasmonic resonance, using the following equation:

$P_{nr}^{An}={2\gamma_{em}^{i}|a_{mode}|}^{2}=2\gamma_{em}^{i}\frac{\left| \kappa_{em} \right|^{2}\left| a_{em} \right|^{2}}{\left( \omega-\omega_{0} \right)^{2}+\left( \gamma_{em}^{r}+\gamma_{em}^{i} \right)^{2}}$ . (S23)

Meanwhile, taking the nano-antenna away, we find that the radiation power of the molecule alone is

$P_{r}^{0}=2\gamma_{0}^{r}\left| a_{em} \right|^{2}.$ (S24)

Therefore, we finally obtain the following two equations

$$\frac{P_{r}}{P_{r}^{0}}=1+\frac{\gamma_{em}^{r}}{\gamma_{r}^{0}}\frac{\left| \kappa_{em} \right|^{2}}{\left( \omega-\omega_{0} \right)^{2}+\left( \gamma_{em}^{r}+\gamma_{em}^{i} \right)^{2}}$$

|  | $\frac{P_{nr}}{P_{r}^{0}}=\frac{\gamma_{abs}}{\gamma_{0}^{r}}+\frac{\gamma_{em}^{i}}{\gamma_{0}^{r}}\frac{\left\vert\kappa_{em} \right\vert^{2}}{\left( \omega-\omega_{0} \right)^{2}+\left( \gamma_{em}^{r}+\gamma_{em}^{i} \right)^{2}}.$ | (S25) |
| --- | --- | --- |

the model the two spectra which can be numerically calculated by FEM simulations (see Eqs. (S10-S12)). Here, we have used a parameter $\gamma_{abs}$to describe all absorption due to the environment that cannot be modelled by the plasmonic resonance Eq. (S23) (i.e., background absorption and the non-resonant part of metallic absorption). Fitting the FEM calculated spectra (Eqs. (S10-S12) with Eqs. (S22) - (S23), we then retrieve all needed parameters ($\omega_{0}, \gamma_{em}^{r}, \gamma_{em}^{i}, \kappa_{em}, \gamma_{abs}$) used in the CMT model.

Inserting Eq. (S25) into Eq. (S9) with the help of Eq. (S14), we obtain the form of $\phi$ in our CMT framework. To get a concise expression of$\phi$, we assume that $\gamma_{0}^{r}\to0$ since the radiation from the low-$\phi$molecule is negligible, and we set the frequency at the resonating one. Under these simplifications, we obtain

|  | $\phi=\frac{\gamma_{em}^{r}}{\gamma_{em}^{r}+\gamma_{em}^{i}+[\gamma_{abs}+\gamma_{0}^{r}\left( \frac{1}{\Phi^{0}}-1 \right)]\frac{{(\gamma_{em}^{r}+\gamma_{em}^{i})}^{2}}{{\kappa_{em}}^{2}}}.$ | (S26) |
| --- | --- | --- |

As a further simplification, we define the effective nonradiative decay rate of the whole system as

|  | $\tilde{\gamma}_{nr}=\gamma_{abs}+\gamma_{0}^{nr}=\gamma_{abs}+\gamma_{0}^{r}\left( \frac{1}{\phi^{0}}-1 \right).$ | (S27) |
| --- | --- | --- |

Thus, Eq. (S26) can be finally simplified as

|  | $\phi=\left( \frac{\gamma_{em}^{r}}{\gamma_{em}^{r}+\gamma_{em}^{i}} \right)\times\left( \frac{1}{1+\tilde{\gamma}_{nr}\frac{\gamma_{em}^{r}+\gamma_{em}^{i}}{{\kappa_{em}}^{2}}} \right),$ | (S28) |
| --- | --- | --- |

which is precisely Eq. (3) in the main text. A phase diagram of the specific case ($\tilde{\gamma}_{\mathrm{nr}}=272 THz$, $\gamma_{\mathrm{em}}^{i}=1.3 \mathrm{THz}$) is shown in Fig. 4d in the main text, where we can see that the $\kappa_{em}$ and $\gamma_{em}^{r}$ has to satisfy a certain relationship to maximize the calculated quantum yield while both $\kappa_{em}$ and $\gamma_{em}^{r}$ are preferred to be large. In order to figure out the requirement of the parameters, we take the partial derivative of $\phi$ respect to $\kappa_{em}$, and set it to zero:

|  | $\frac{\partial\phi}{\partial\kappa_{em}}=\frac{2\tilde{\gamma}_{nr}\gamma_{em}^{r}}{{(1+\tilde{\gamma}_{nr}\frac{\gamma_{em}^{r}+\gamma_{em}^{i}}{{\kappa_{em}}^{2}})}^{2}{\kappa_{em}}^{3}}=0.$ | (S29) |
| --- | --- | --- |

By solving Eq. S29, we get the extreme point for each given $\kappa_{em}$, which satisfies:

|  | $\kappa_{em}=\sqrt{\frac{\tilde{\gamma}_{nr}}{\gamma_{em}^{i}}({\gamma_{em}^{r}}^{2}-{\gamma_{em}^{i}}^{2})}.$ | (S30) |
| --- | --- | --- |

For the specific region where $\gamma_{em}^{r}\gg\gamma_{em}^{i}$, Eq. S30 can be simplified as:

|  | $\kappa_{em}=\sqrt{\frac{\tilde{\gamma}_{nr}}{\gamma_{em}^{i}}}\gamma_{em}^{r},$ | (S31) |
| --- | --- | --- |

which is the cyan dashed line shown in Fig. S12. It is obvious that the parameter $\phi$ can be maximized only if we enlarge $\kappa_{em}$ and $\gamma_{em}^{r}$ while making the ($\kappa_{em}, \gamma_{em}^{r}$) point in the phase diagram as close to the cyan line as possible. For the realistic case, we show in Fig. S10b the dependence of $\kappa_{exc}$ and $\gamma_{exc}^{r}$ as the structural asymmetry increases along the white dashed in Fig. S12. The above understanding leads us to the reason why we can increase the calculated quantum yield $\phi$ by increasing the degree of asymmetry for the corresponding system: we are actually pushing the system closer to the cyan line by decreasing $\gamma_{em}^{r}$ and leaving $\kappa_{em}$ almost the same, resulting in better performance even that smaller $\gamma_{em}^{r}$ is not favored. Similar effect can be seen in the black dashed line in Fig. S12, where we press the point towards the cyan line by enlarging $\kappa_{em}$ and setting $\gamma_{em}^{r}$ fixed.


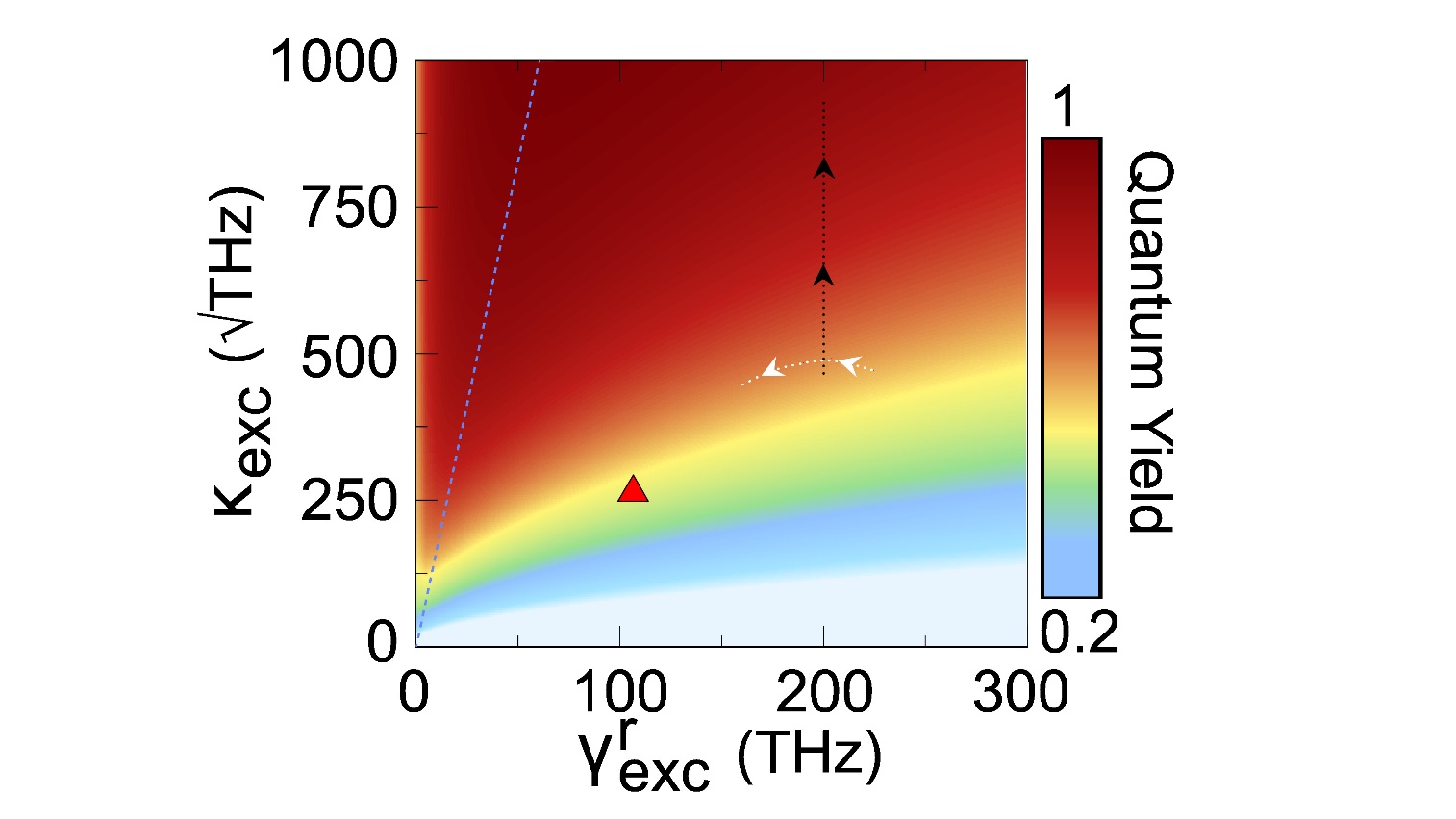


**Figure S12:** detailed analyses for the phase diagram in Fig. 4d in the main text. The white dashed line, black dashed line and the red triangle are the same as that in Fig. 4d in the main text. The cyan dashed line is the calculated local optima for each given $\kappa_{em}$.

**Section 7**– **Retrieving CMT parameters from full-wave simulations**

We describe how to get the CMT parameters for those structures studied in Fig. 4 (main text). Here, we choose two particular nano-antenna structures as two examples to illustrate our fitting procedures in excitation and emission processes. As shown in Figs. S13a-S13c, red lines represent the numerically computed spectra of reflection (*R*), spectra of absorption (*A*), and field-enhancement ($F_{exc})$for the sample containing a periodic array of the nano-antenna with $b_{1}=150 \mathrm{nm}$, $b_{2}=229 \mathrm{nm}$, obtained using the simulation methodology described in Sec. 5 in SI. Via tuning the CMT parameters for excitation process, we find that the set of parameters $(\kappa_{exc}=963 \sqrt{\mathrm{THz}}, d_{exc}^{0}=2.28 \sqrt{\mathrm{THz}},\gamma_{exc}^{r}=75.7 THz, \gamma_{exc}^{i}=8.20 \mathrm{THz})$can yield the best-fitted CMT curves, which are shown in the same figures as blue lines, computed by Eqs. (S18-S20). We note that the agreement between CMT and FEM spectra (particularly the *R* spectrum) are not good at frequencies lower that the resonance frequency. This is because we only considered a single plasmonic mode in our CMT model and assumed that all parameters are frequency nondispersive. In reality, however, tails of other mode(s) located at low-frequency regime can inevitably influence the spectra of this mode. Similarly, Figs. S13d-S13e compare the numerically simulated and CMT-fitted spectra of ${P_{r}}/{P_{r}^{0}}$ and ${P_{nr}}/{P_{r}^{0}}$or the sample containing a periodic array of the nano-antenna with $b_{1}=150 \mathrm{nm}$, $b_{2}=196 \mathrm{nm}$, calculated using Eq. (S25), respectively. CMT parameters yielding the best-fitted spectra are following combinations: $(\kappa_{em}=489 \sqrt{\mathrm{THz}},\gamma_{em}^{r}=198.0 THz, \mathrm{and}\gamma_{em}^{i}=1.20 \mathrm{THz})$.


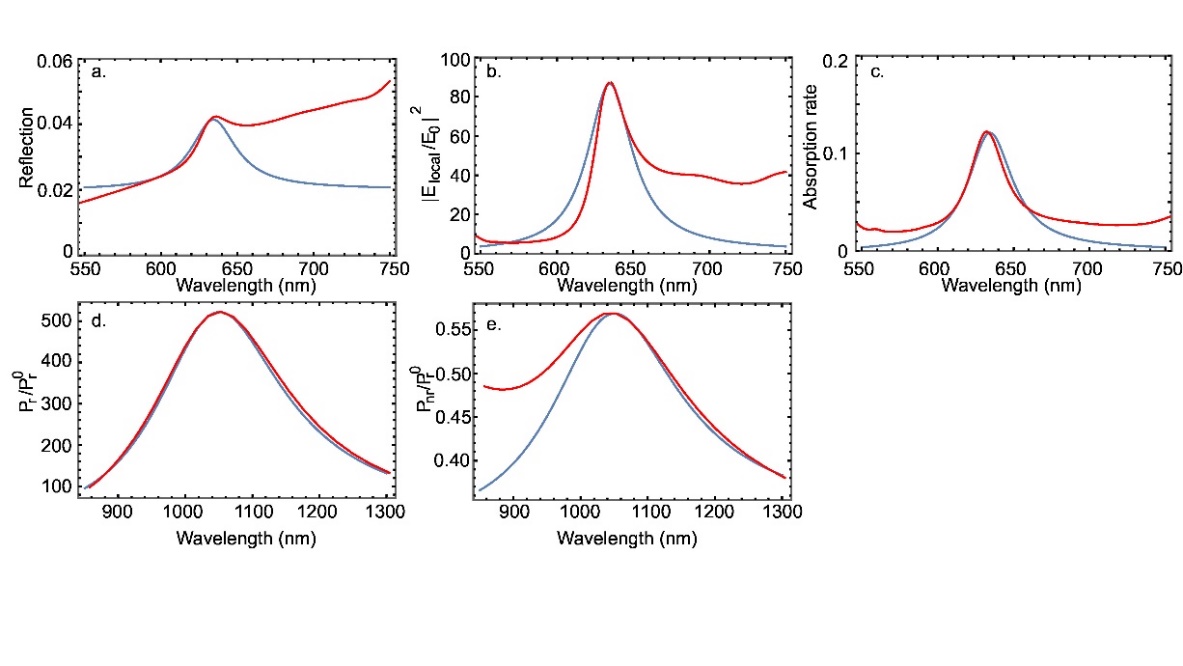


**Figure S13**. Simulated (red) and CMT-calculated (blue) spectra of **a.** reflection, **b.** absorption, and **c.** ${|E_{local}/E_{\boldsymbol{0}}|}^{2}$in the excitation process and **d.**${P_{r}}/{P_{r}^{0}}$, e.${P_{nr}}/{P_{r}^{0}}$ in the emission process. Structural parameters of the sample studied in **a-c** are $b_{1}=150 \mathrm{nm}$ and $b_{2}=229 \mathrm{nm}$, while those of the sample studied in **d-e** are $b_{1}=150 \mathrm{nm}$ and $b_{2}=196 \mathrm{nm}$.

Based on the fitting procedures mentioned above, we finally obtain the CMT parameters for all nano-antennas located on the blue line in Fig. 4a and on the white line in Fig. 4b in the main text. Table S4 and S5 contain, respectively, the values of CMT parameters of these two series of structures, for two processes.

**Table S4**. Retrieved excitation-process CMT parameters for the series of nano-antennas located on the blue line in Fig. 4a. $b_{1}$ and $b_{2}$ are the bar lengths of the nano-antennas.

| **Sample number** | $\boldsymbol{b}_{\boldsymbol{1}}$ **(nm)** | $\boldsymbol{b}_{\boldsymbol{2}}$ **(nm)** | $\boldsymbol{\kappa}_{\boldsymbol{exc}}$ **(**$\sqrt{\mathrm{THz}}$**)** | $\boldsymbol{\gamma}_{\boldsymbol{exc}}^{\boldsymbol{r}}$ **(THz)** | $\boldsymbol{\gamma}_{\boldsymbol{exc}}^{\boldsymbol{i}}$ **(THz)** | $\boldsymbol{d}_{\boldsymbol{exc}}^{\boldsymbol{0}}$ **(**$\sqrt{\mathrm{THz}}$**)** |
| --- | --- | --- | --- | --- | --- | --- |
| 1 | 120 | 232 | 845 | 37.6 | 6.66 | 2.26 |
| 2 | 130 | 231 | 935 | 51.1 | 7.60 | 2.27 |
| 3 | 140 | 230 | 958 | 63.5 | 7.80 | 2.28 |
| 4 | 150 | 229 | 963 | 75.7 | 8.20 | 2.29 |
| 5 | 160 | 228 | 935 | 85.7 | 8.00 | 2.30 |
| 6 | 170 | 227 | 885 | 93.6 | 7.60 | 2.31 |
| 7 | 180 | 226 | 831 | 100.0 | 7.60 | 2.32 |
| 8 | 190 | 225 | 779 | 107.5 | 7.41 | 2.33 |
| 9 | 200 | 224 | 713 | 113.6 | 7.41 | 2.34 |

**Table S5.** Retrieved emission-process CMT parameters for the series of nano-antennas located on the white line in Fig. 4b. $b_{1}$ and $b_{2}$ are the bar lengths of the nano-antennas.

| **Sample number** | $\boldsymbol{b}_{\boldsymbol{1}}$ **(nm)** | $\boldsymbol{b}_{\boldsymbol{2}}$ **(nm)** | $\boldsymbol{\kappa}_{\boldsymbol{em}}$ **(**$\sqrt{\mathrm{THz}}$**)** | $\boldsymbol{\gamma}_{\boldsymbol{em}}^{\boldsymbol{r}}$ **(THz)** | $\boldsymbol{\gamma}_{\boldsymbol{abs}}$ $\mathbf{(THz)}$ | $\boldsymbol{\gamma}_{\boldsymbol{em}}^{\boldsymbol{i}}$ **(THz)** |
| --- | --- | --- | --- | --- | --- | --- |
| 1 | 50 | 202.5 | 440 | 156.6 | 66.1 | 1.34 |
| 2 | 60 | 202.5 | 447 | 160.6 | 66.1 | 1.34 |
| 3 | 70 | 202.5 | 455 | 163.8 | 66.1 | 1.34 |
| 4 | 80 | 202.5 | 460 | 167.4 | 66.1 | 1.34 |
| 5 | 90 | 201.5 | 467 | 171.1 | 66.1 | 1.34 |
| 6 | 100 | 201 | 473 | 174.8 | 66.1 | 1.34 |
| 7 | 110 | 200.5 | 475 | 178.6 | 66.1 | 1.34 |
| 8 | 120 | 199.5 | 478 | 182.4 | 66.1 | 1.28 |
| 9 | 130 | 198.5 | 480 | 186.2 | 66.1 | 1.25 |
| 10 | 140 | 197 | 484 | 190.1 | 66.1 | 1.22 |
| 11 | 150 | 196 | 489 | 198.0 | 66.1 | 1.20 |
| 12 | 156 | 195 | 492 | 204.0 | 66.1 | 1.16 |
| 13 | 162 | 193 | 492 | 211.2 | 66.1 | 1.16 |
| 14 | 168 | 190.5 | 489.5 | 216.3 | 66.1 | 1.13 |
| 15 | 174 | 188 | 484 | 222.6 | 66.1 | 1.08 |
| 16 | 180 | 186 | 471 | 224.7 | 66.1 | 1.05 |

**Section 8** **– CMT parameters of those additional structures studied in Fig. 4(d)**

Following exactly the same fitting procedures, we retrieved all CMT parameters for those nano-antennas on the black vertical line depicted in Fig. 4d in the main text. The structure parameters and the retrieved CMT parameters are shown in Table S6. The simulated enhancement of excitation and quantum yield are shown in Fig. S14

Finally, we also retrieved the CMT parameters of an *undesigned* nano-antenna represented by the red triangle in Fig. 4d. Fig. S15 schematically depicts the mode profile of the resonance supported by such an antenna, and its structural parameters and retrieved CMT parameters are shown in Table S7.

**Table S6.** Retrieved emission-process CMT parameters for the series of nano-antennas located on the black dashed line in Fig. 4d. $b_{1}$ and $b_{2}$ are the bar lengths of the nano-antennas，d is the gap size (distance) between bars.

| **Sample number** | $\boldsymbol{b}_{\boldsymbol{1}}$ **(nm)** | $\boldsymbol{b}_{\boldsymbol{2}}$ **(nm)** | $\boldsymbol{\kappa}_{\boldsymbol{em}}$ **(**$\sqrt{\mathrm{THz}}$**)** | $\boldsymbol{\gamma}_{\boldsymbol{em}}^{\boldsymbol{r}}$ **(THz)** | $\boldsymbol{\gamma}_{\boldsymbol{abs}}$ $\mathbf{(THz)}$ | $\boldsymbol{\gamma}_{\boldsymbol{em}}^{\boldsymbol{i}}$ **(THz)** | $\boldsymbol{d}$ **(nm)** |
| --- | --- | --- | --- | --- | --- | --- | --- |
| 1 | 150 | 202 | 462 | 198.0 | 66.1 | 1.20 | 80 |
| 2 | 150 | 201 | 463 | 198.0 | 66.1 | 1.16 | 70 |
| 3 | 150 | 200 | 466 | 198.0 | 66.1 | 1.20 | 60 |
| 4 | 150 | 198 | 475 | 198.0 | 66.1 | 1.02 | 50 |
| 5 | 150 | 196 | 489 | 198.0 | 66.1 | 1.20 | 40 |
| 6 | 140 | 191 | 535 | 198.0 | 66.1 | 1.17 | 30 |
| 7 | 130 | 184 | 633 | 198.0 | 66.1 | 1.62 | 20 |
| 8 | 110 | 175 | 927 | 198.0 | 66.1 | 1.60 | 10 |

**Table S7.** Retrieved emission-process CMT parameters of the undesigned nan-antenna represented by the red triangle in Fig. 4 d in the main text.

| **Sample number** | $\boldsymbol{b}_{\boldsymbol{1}}$ **(nm)** | $\boldsymbol{b}_{\boldsymbol{2}}$ **(nm)** | $\boldsymbol{\kappa}_{\boldsymbol{em}}$ **(**$\sqrt{\mathrm{THz}}$**)** | $\boldsymbol{\gamma}_{\boldsymbol{em}}^{\boldsymbol{r}}$ **(THz)** | $\boldsymbol{\gamma}_{\boldsymbol{em}}^{\boldsymbol{i}}$ **(THz)** | $\boldsymbol{d}$ **(nm)** |
| --- | --- | --- | --- | --- | --- | --- |
| 1 | 227.5 | 227.5 | 263 | 106.6 | 1.55 | 40 |


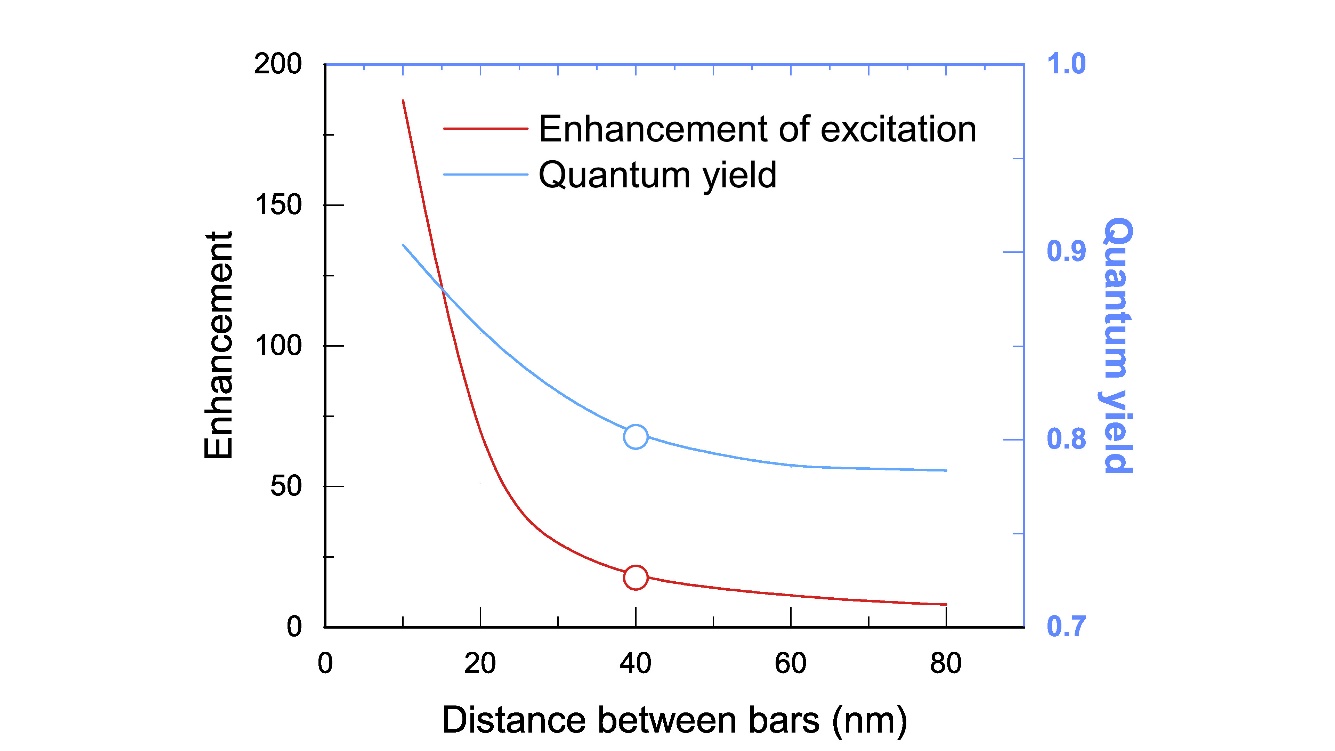


**Figure S14.** Simulated excitation enhancement (red curve, left axis) and quantum yield (blue curve, right axis) for the additional structures located on the black vertical line in Fig. 4d in the main text. Hollow circles denote the structure realized experimentally.


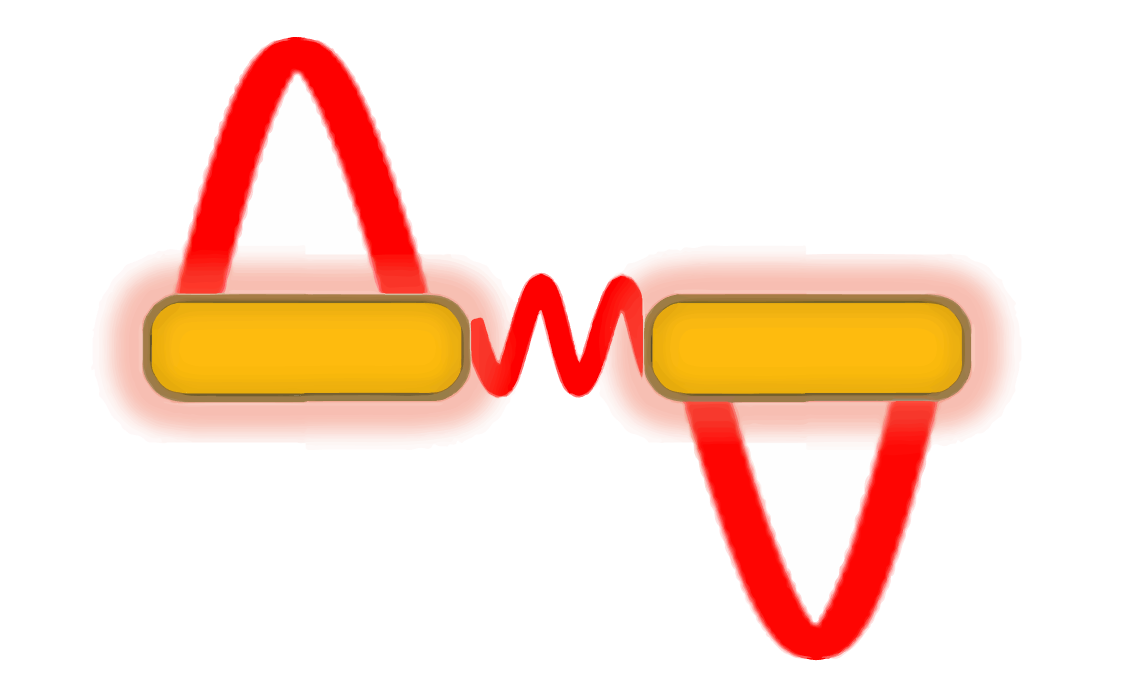


**Figure S15:** Schematics of resonance mode supported by the nano-antenna represented by the red triangle in Fig. 4d in the main text.

**Section 9 – Bleaching time of AIEE1000 on glass and antenna**

We measured the bleaching time of AIEE1000 molecules on antenna and on bare-glass respectively. For AIEE1000 molecules on antenna, the obvious step-like intensity drop helps to determine the bleaching time $t_{B}$ (as shown in Fig. S16a). Fitting $t_{B}$ histogram with an exponential decay, we could get the bleaching time value representing the time for ($1-e^{-1}$) of all molecules bleached at a certain condition. While for molecules on bare-glass, nearly no intensity drop can be distinguished from the intensity-time trajectory. Therefore, we measured the intensity difference $\delta I$ between two consecutive frames and divided it by the referential single molecule intensity $I_{0}$ ($I_{0}=5.304 \mathrm{cpf}$, measurement described in Sec. 4 of SI), then number of molecules ($n$) bleached in this time-point can be estimated. Plotting the $n-t_{B}$ data into histograms, we could further get the bleaching time similarly. Bootstrapping was carried out to avoid experimental errors.

**
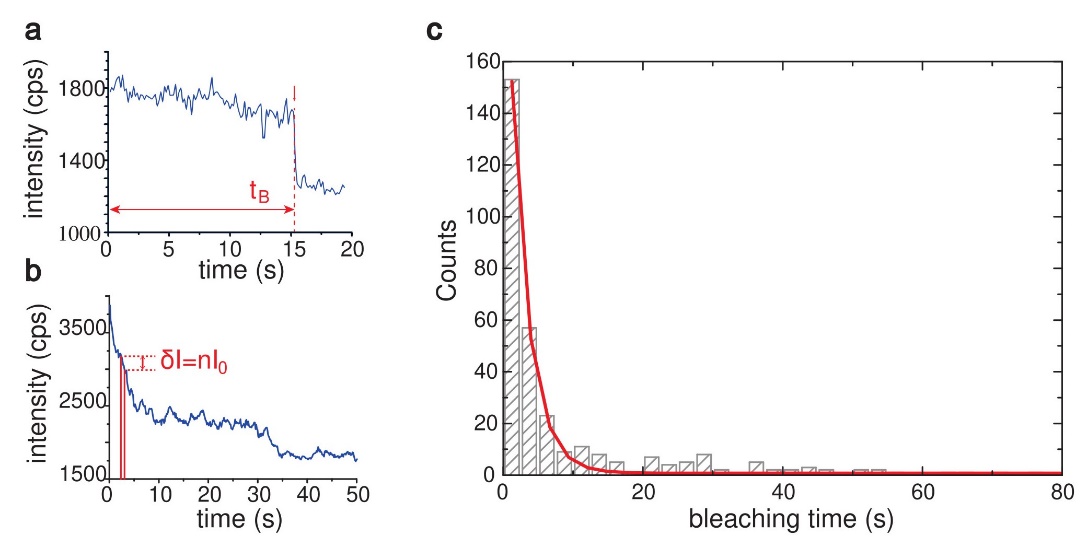
**

**Figure S16:** Calculation of bleaching time $\boldsymbol{t}_{\boldsymbol{B}}$. **a.** Typical intensity-time trace of single AIEE1000 molecule near an antenna, the digital (step-like) sudden drop at 15^th^ second represents a photobleaching process; **b.** Typical intensity-time trace of AIEE1000 molecules on bare-glass, $\delta I$ comes from intensity difference between two consecutive frames; **c.** Typical statistical distribution of $\boldsymbol{t}_{\boldsymbol{B}}$ from AIEE1000 molecules near DB-antenna (150-250 nm antenna, grey histogram), red-curve and inset table shows 1-exponential fit of $\boldsymbol{t}_{\boldsymbol{B}}$-distribution, calculated bleaching time is 2.62±0.06 s.

We increased the 633nm laser intensity in gradient and measured the corresponding bleaching times respectively. For clarity, we use the bleaching rate to evaluate the effect of excitation intensity on molecules bleaching process. As shown in Fig. S17, when increase the excitation intensity, bleaching process is accelerated linearly, meaning molecules at different excitation intensity emits relatively constant number of photons before bleaching. The variations of calculated total photons (grey-columns in Fig. S17) may come from error of measurements of excitation power and the non-uniformity of molecules distribution on glass.


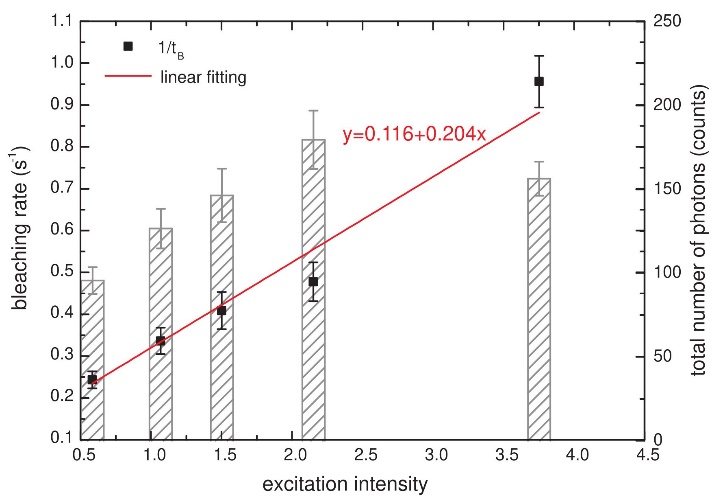


**Figure S17:** Bleaching rate changes with excitation intensity. Black-squares represent bleaching rates (${t_{B}}^{-1}$) calculated from data in SI-6 with different excitation intensity; grey-columns are products of bleaching time multiplied by intensity respectively, representing the total photons emitted from one molecule at different excitation intensity; both error bars come from standard deviations of bootstrapped $\boldsymbol{t}_{\boldsymbol{B}}$-results.

**References:**

1. Melhuish, W. H. Quantum Efficiencies of Fluorescence of Organic Substances: Effect of Solvent and Concentration of the Fluorescent Solute1. *The Journal of Physical Chemistry* **65**, 229-235 (1961).

2. Casalboni, M., De Matteis, F., Prosposito, P., Quatela, A. & Sarcinelli, F. Fluorescence Efficiency of Four Infrared Polymethine Dyes. *Chem. Phys. Lett.* **373**, 372-378 (2003).

3. Qian, G. et al. Band Gap Tunable, Donor− Acceptor− Donor Charge-Transfer Heteroquinoid-Based Chromophores: Near Infrared Photoluminescence and Electroluminescence. *Chem. Mater.* **20**, 6208-6216 (2008).

4. Bharadwaj, P. & Novotny, L. Spectral Dependence of Single Molecule Fluorescence Enhancement. *Opt. Express* **15**, 14266-14274 (2007).
